# Differential response variability of black-capped chickadees to wingbeat sounds and vocalizations

**DOI:** 10.1242/bio.062598

**Published:** 2026-06-24

**Authors:** Prateek K. Sahu, Kailyn Nottebrock, Julian Ratch, Sarah M. L. Smeltz, Katharine H. Stenstrom, Moriah J. Deimeke, Christopher B. Sturdy

**Affiliations:** ^1^ Department of Psychology, University of Alberta, Edmonton T6G 2R3, Canada; ^2^ Neuroscience and Mental Health Institute, University of Alberta, Edmonton T6G 2R3, Canada

**Keywords:** Acoustic cues, Wingbeats, Threat assessment, Cue integration

## Abstract

Prey species use various cues (visual, acoustic, olfactory) to detect approaching predators. Among these, visual cues are generally considered reliable, providing immediate and accurate information about potential dangers. While acoustic cues also play a key role in threat assessment in birds, the relative importance of different auditory cues and their integration are less well studied. Here, we investigated how black-capped chickadees (*Poecile atricapillus*) respond to predator and non-predator wingbeats and vocalizations presented independently and in sequence with each other. In an exploratory playback experiment with 16 chickadees, we found no differences in how chickadees responded to predator versus non-predator acoustic cues when presented individually or sequentially. However, sequential presentations of acoustic cues had reduced response variance compared to solo playbacks. These findings suggest that black-capped chickadees may lack specific recognition mechanisms for discriminating predator from non-predator wingbeats in isolation but may integrate multiple cues to improve threat assessment reliability. Our results demonstrate the potential role of non-vocal cues in acoustic threat assessment in small passerines and the importance of considering within-modality cue integration in anti-predator behaviour studies. Future behavioural study designs would benefit from incorporating the current findings.

## Teaser

**Summary:** Black-capped chickadees, a North American songbird, appear to show stronger response with reduced variance, when acoustic cues (wingbeat or vocalizations sound) are presented sequentially as compared to when presented in isolation. The study highlights the importance of non-vocal cues in acoustic threat assessment.

## INTRODUCTION

Prey species can use a wide range of cues, such as visual, auditory, and chemical cues to detect approaching predators. Prey animals collect, evaluate, and integrate threat-related information differently based on their environment and the information that is most advantageous for their survival. While some species benefit from combining information from several sources to produce the most accurate assessment of the current threat (Caballero-Díaz et al., 2023; Lukas et al., 2021), other species prioritize different modalities in a hierarchical manner (Arteaga-Torres et al., 2020). The effectiveness of these strategies is shaped by evolutionary pressures, where prey species can develop specialized sensory adaptations for their survival (Caro, 2005). Environmental variability can determine which sensory modalities would provide the most reliable information regarding potential threats (Smith and Evans, 2013). Understanding anti-predator behaviour requires integration of both field and laboratory studies: field experiments provide ecological validity by assessing threat responses in natural contexts, and laboratory experiments enable precise stimulus control to isolate specific sensory and decision-making mechanisms.

Prey animals should balance the costs and benefits of their behavioural response (low to high) to predator cues, depending on the severity of threat and certainty associated with the threat (Caro, 2005; Crane et al., 2024; Munoz and Blumstein, 2012). Variation in response would also depend on the sensory modality of the predator signal (e.g. visual cues can provide more certainty for the presence of proximal threat). The optimal response to threat may depend on the integration of multisensory stimuli or the choice of integrating multisensory information, and an individual's internal state and surrounding environment (Munoz and Blumstein, 2012, 2020). A recent meta-analysis found no evidence that modality of information or combination of cue modalities about predation risk in experimental conditions influenced mean magnitude response in birds (Mathot et al., 2024). Birds' responses to multimodal cues had lower among-study variance compared with the response to unimodal cues, which could be explained by the theory of maximum-likelihood estimation (MLE) integration (Mathot et al., 2024). For example, under MLE integration, when both visual and auditory cues are presented together in an experiment, we would expect reduction in variance as compared to unimodal cues (Ernst and Banks, 2002; Mathot et al., 2024). But how would the pattern of response change with different threat levels of unimodal cues? In the auditory modality, we would expect higher responses to mobbing calls as compared to predator vocalizations, as mobbing calls can provide more information about threat levels than predator produced vocalizations. The ultimate response would depend on the type of information gleaned from those auditory cues and the certainty associated with those cues.

While predators often approach stealthily during an attack, this may not always be possible, resulting in a variety of non-vocal cues. Birds' sensitive auditory perception might enable them to detect and respond to these non-vocal cues (Shearer and Beilke, 2023); however, little work has been done on how birds respond to non-vocal cues. For example, wing-flapping of birds may indicate approaching threat (Hingee and Magrath, 2009). In scaled doves (*Columbina squammata*), who have specialized feathers, the probability of exhibiting anti-predator behaviour was higher after hearing a conspecific wing trill as compared to conspecific or heterospecific vocalizations (Amorim and Dias, 2021). Sparrows (Passerellidae) responded similarly to both predator (hawk) and non-predator (passerine) wingbeats in a field playback experiment (Shearer and Beilke, 2023). It is not clear how reliable and important wingbeat stimuli are compared to direct information (such as predator or non-predator vocalizations). Wingbeats alone may not contain certainty information about presence of threat but associated with predator vocalizations (context specific) may provide more information about the predator (Barrera et al., 2011; Munoz and Blumstein, 2012). Under MLE integration, when both wingbeats and predator vocalizations are presented together, we would expect lower variance in response as compared to wingbeats or predator vocalization presented alone.

**Fig. 1.**
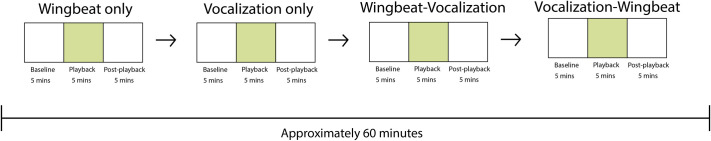
**Experimental procedure.** Schematics showing procedure for sequence 1 from Table 1. Rest of the procedures were followed similarly.

To understand the differential response to wingbeats and predator vocalizations, we used black-capped chickadees (*Poecile atricapillus*) in a laboratory-based exploratory playback study. Black-capped chickadees are a North American passerine who form flocks throughout the year outside the breeding season. Within these flocks, chickadees can signal predator threat with mobbing calls (Smith, 1991). Chickadees obtain and respond to threat information such as size, type, and degree of threat through acoustic cues (Congdon et al., 2020; Templeton et al., 2005). For example, birds produce high number of ‘chick-a-dee’ calls and D notes in response to a high threat predator versus a low threat predator (Templeton et al., 2005). Playback studies show chickadees produce more anti-predator behaviour (e.g. produce more ‘chick-a-dee’ calls) in response to predator vocalizations as compared to non-predator vocalizations (Congdon et al., 2016). Apart from vocalizations, chickadees show freezing, taking cover, and increased scanning or movement behaviour in response to predator threat. Here, we asked whether black-capped chickadees respond differentially to predator and non-predator wingbeats and vocalizations, and whether sequential presentation of these acoustic cues produces additive effects (Fig. 1). We predicted that chickadees would display stronger anti-predator responses to predator species vocalization compared to non-predator species vocalization. Further, we hypothesized that sequential presentation of unimodal cues (predator vocalization and wingbeat) would increase threat certainty, thereby eliciting stronger anti-predator responses compared to solo cue presentations.

**Table 1.** Each row sequence (seq.) of playback groups (two birds per row)

Seq.	Predator group (*N*=8)				Non-predator group (*N*=8)			
1	WO	VO	WV	VW	WO	VO	WV	VW
2	WO	VO	VW	WV	WO	VO	VW	WV
3	VO	WO	WV	VW	VO	WO	WV	VW
4	VO	WO	VW	WV	VO	WO	VW	WV

## RESULTS

### Activity response

Birds in the predator group responded with fewer perch hops [mean=186.11, s.d.=164.97, range: 0-537; evidence ratio=9.02 (moderate), posterior probability of reduction=90%] than those in the non-predator condition (mean=234.69, s.d.=117.74, range: 0-488), but with wide uncertainties (posterior mean=0.57, 95% CrI: 0.27-1.18; ∼73% fewer to 18% more perch hops). We did not find evidence that birds in both groups had different responses (evidence ratio=2.02, posterior probability of reduction=67%) across playback phases (baseline: 216.06±140.85, playback: 213.10±145.05, post-playback: 202.04±150.83).

**Fig. 2.**
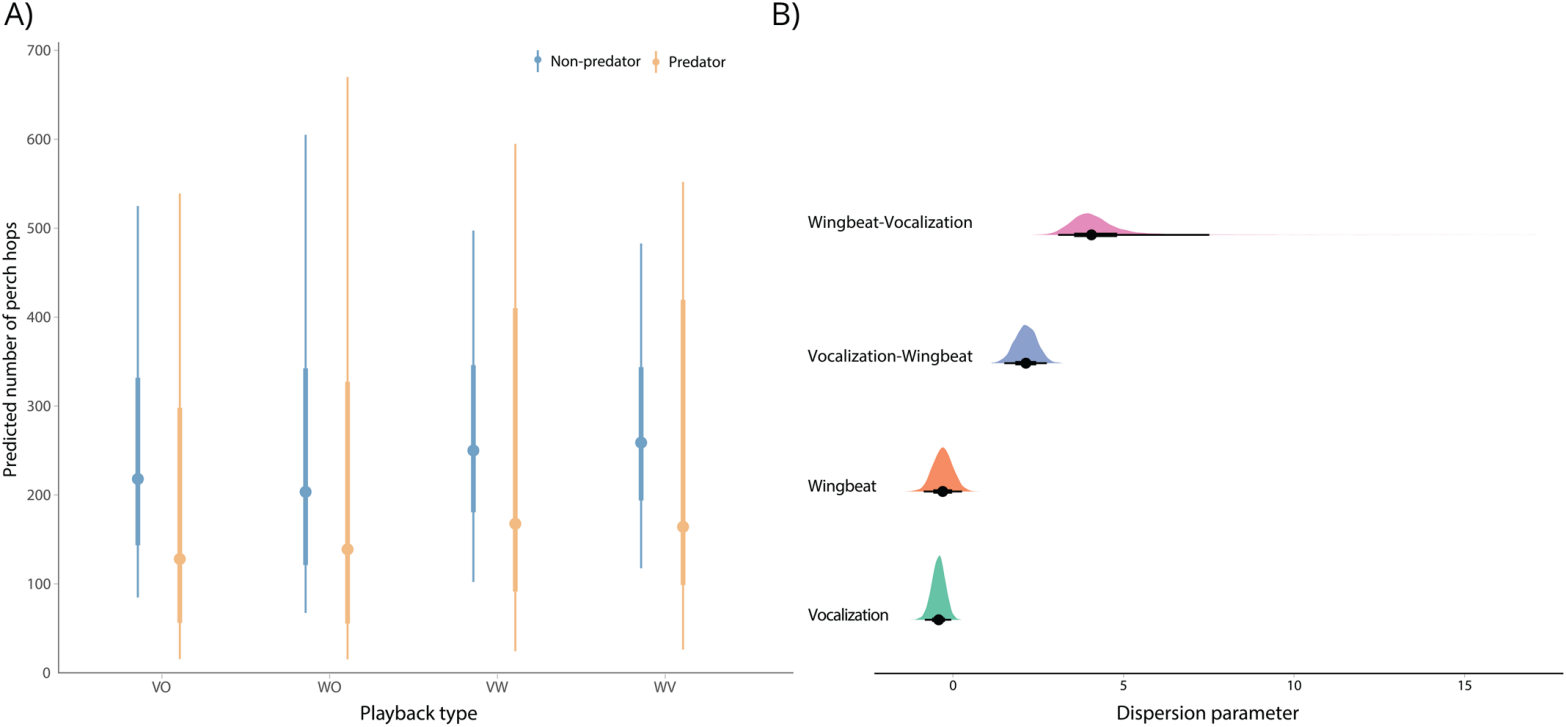
**(A) Predicted number of perch hops by playback type and experimental condition.** Points represent posterior means with 66% (thick lines) and 95% (thin lines) credible intervals from a Bayesian negative binomial mixed-effects model (*N*=8 for each non-predator and predator). Birds in the predator group (amber) showed reduced perch hop activity compared to the non-predator group (slate blue) across all playback types. Playback types: VO, vocalization only; WO, wingbeat only; VW, sequential vocalization-wingbeat; WV, sequential wingbeat-vocalization. (B) Posterior distributions of the dispersion parameter in log-scale by playback type for perch hop response. Half-eye plots show posterior density distributions with thick lines representing 66% credible intervals, thin lines representing 95% credible intervals, and black points indicating posterior medians (*N*=16 for each playback type). Sequential playback types showed higher dispersion parameter values than solo playback types, indicating reduced overdispersion. Higher dispersion parameter values correspond to lower variability.

Among stimulus playback types (combined both predator and non-predator groups), WO produced 187.58±160.22 (range: 0-537) perch hops whereas VO produced 170.27±138.04 (range: 0-534) perch hops. In sequential playback types, VW produced 235.52±138.54 (range: 3-506) perch hops and WV produced 248.25±131.27 (range: 25-512) perch hops (Fig. S1). We found no differences for perch hops to combined playback types (VW and WV) compared to solo playback types (VO and WO) (evidence ratio=2.37, posterior probability of increase=70%, posterior mean=1.30, 95% CrI: 0.54-2.96; 46% fewer to 196% more responses; Fig. 2A; Table S1).

We tested whether solo playback types (VO and WO) have wider variance as compared to combined playback types (VW and WV). We modelled and compared the distributional parameter for the playback stimuli type. Sequential combined playback types (VW and WV) showed substantially higher dispersion parameter values than solo playback types (WO and VO) [evidence ratio>9999 (or Inf), estimate (natural scale)=854.05, 95% CrI (natural scale): 247.15-5166.75; posterior probability>99%], indicating less overdispersion response patterns during sequential presentations (higher dispersion parameter values indicate reduced variability in responses; Fig. 2B). The sequential combined playback types had relatively more uncertainty in estimating the variance (natural scale) (VW: 8.50, 95% CrI: 4.53-15.64 and WV: 75.19, 95% CrI: 21.76-1844.57) than solo playback types (VO: 0.66, 95% CrI: 0.44-0.95 and WO: 0.74, 95% CrI: 0.42-1.31).

### Vocal response

#### Tseet calls

Birds in the predator group produced 24.72±41.2 (mean±s.d.; range: 0-202) ‘tseet’ calls while those in the non-predator group produced 21.52±31.46 (range: 0-147) tseet calls. Provided our Bayesian mixed-effects negative binomial model is a good approximation, we did not find evidence that birds in the predator group produced different number of tseet calls compared to those in the non-predator group (evidence ratio=0.85, posterior probability of increase=46%) with wide uncertainties (posterior mean=0.91, 95% CrI: 0.23-3.56). Overall, in the playback phase, birds produced 19.42±34.87 (range: 0-202) tseet calls as compared to 21.68±32.79 (range: 0-153) tseet calls in the baseline phase and 28.25±41.54 (range: 0-147) tseet calls in the post-playback phase.

**Fig. 3.**
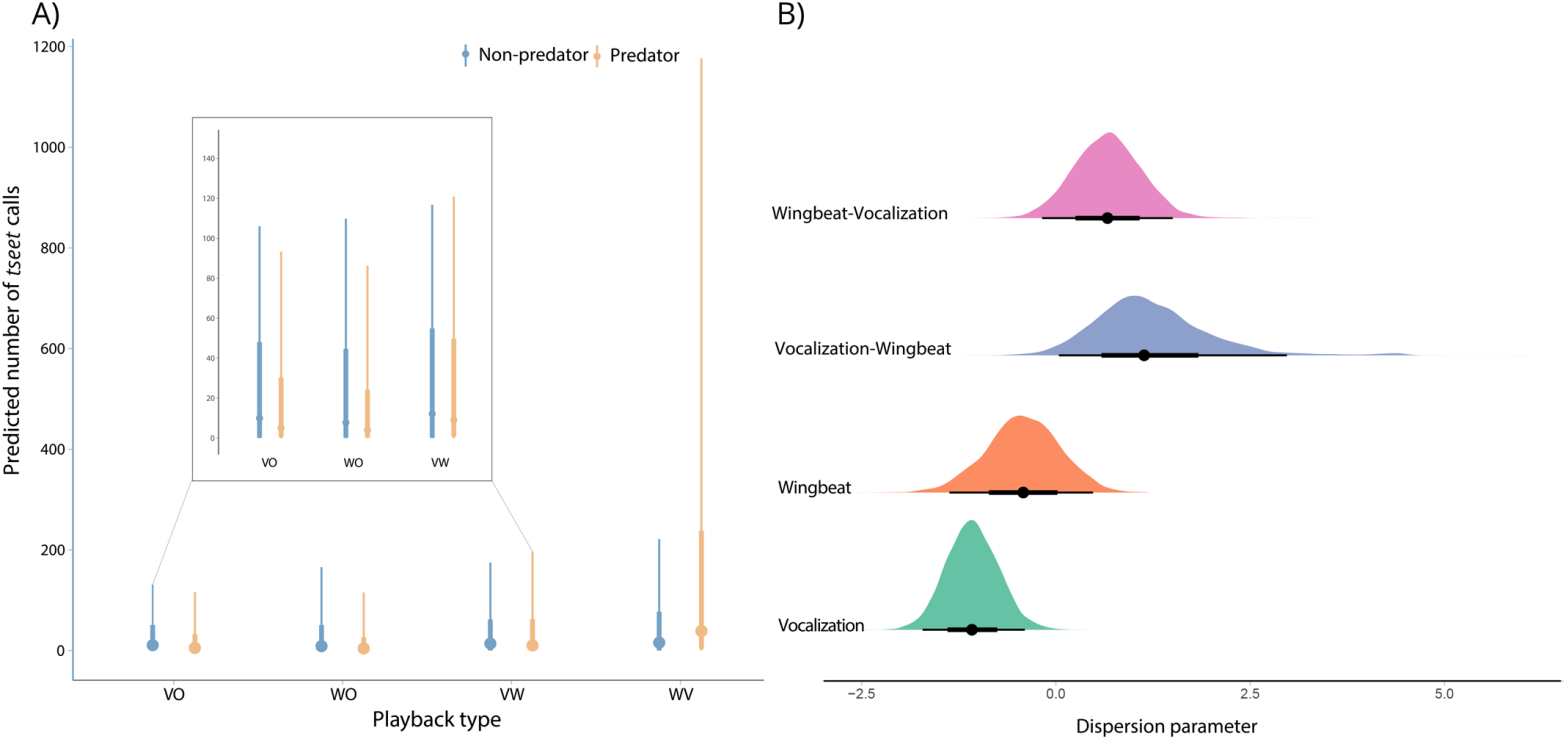
**(A) Predicted number of tseet calls by playback type and experimental condition.** Points represent posterior means with 66% (thick lines) and 95% (thin lines) credible intervals from a Bayesian negative binomial mixed-effects model (*N*=8 for each non-predator and predator). Birds in the predator group (amber) showed reduced perch hop activity compared to the non-predator group (slate blue) across all playback types. The inset graph shows a zoomed-in view of VO, WO, and VW. Playback types: VO, vocalization only; WO, wingbeat only; VW, sequential vocalization-wingbeat; WV, sequential wingbeat-vocalization. (B) Posterior distributions of the dispersion parameter in log-scale by playback type for tseet call responses. Half-eye plots show posterior density distributions with thick lines representing 66% credible intervals, thin lines representing 95% credible intervals, and black points indicating posterior medians (*N*=16 for each playback type). Sequential playback types showed higher dispersion parameter values than solo playback types, indicating reduced overdispersion during sequential presentations. Higher dispersion parameter values correspond to lower variability.

Among stimulus playback types (combined both predator and non-predator groups), WO produced 9.64±17.7 (range: 0-70) tseet calls whereas VO produced 18.62±33.67 (range: 0-147) tseet calls. In sequential playback types, VW produced 26.54±35.04 (range: 0-133) tseet calls and WV produced 37.66±48.29 (range: 0-202) tseet calls (Fig. S2). We found no evidence that birds produced different number of tseet calls during sequential playback types compared to solo playback types (evidence ratio=2.14, posterior probability of increase=68%) with wide uncertainties (posterior mean=1.46, 95% CrI: 0.38-5.42; 62% fewer to 442% more calls, Fig. 3A; Table S2).

We also tested whether solo playback types (VO or WO) induced wider variance as compared to combined playback types (VW or WV). Sequential playback types showed substantially higher dispersion parameter values than solo playback types for tseet calls [evidence ratio=2665.67, estimate (natural scale)=10.18, 95% CrI (natural scale): 3.22-35.51; posterior probability=100%], indicating less overdispersion calling patterns during sequential presentations (Fig. 3B). The sequential combined playback types had slightly more uncertainty in estimating the variance (natural scale) (VW: 3.42, 95% CrI: 1.04- 19.49 and WV: 1.93, 95% CrI: 0.84-4.48) than solo playback types (VO: 0.34, 95% CrI: 0.18-0.67 and WO: 0.66, 95% CrI: 0.25-0.1.62).

#### Chick-a-dee calls

**Fig. 4.**
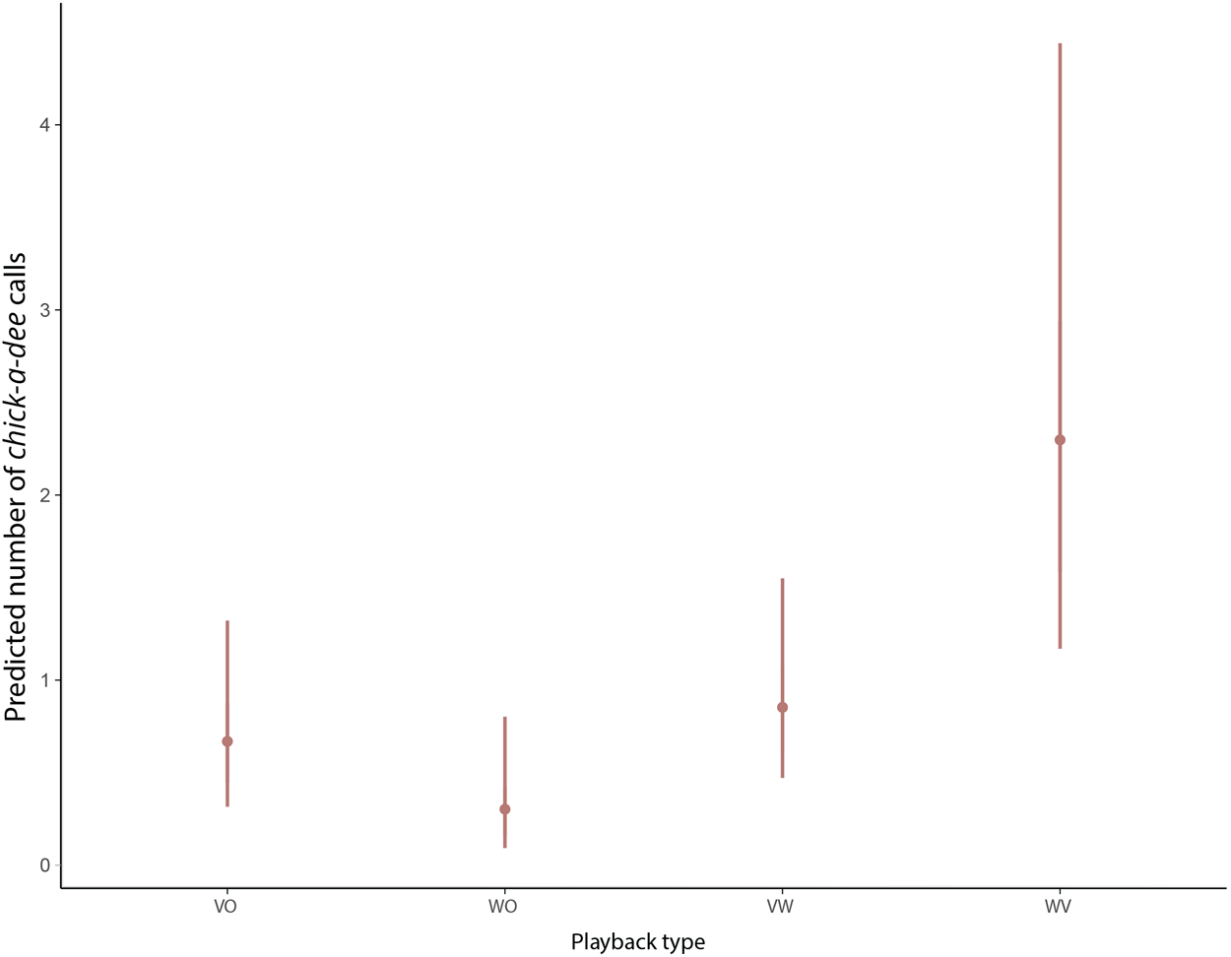
**Predicted number of chick-a-dee calls by playback type from marginal means analysis.** Points represent posterior medians with 95% highest posterior density (HPD) intervals from a Bayesian negative binomial hurdle model (*N*=16 for each playback type). The WV playback type (wingbeat-vocalization sequence) elicited substantially more chick-a-dee calls with high uncertainty than other playback types. Playback types: VO, vocalization only; WO, wingbeat only; VW, vocalization-wingbeat sequence; WV, wingbeat-vocalization sequence.

Approximately 70% of observations recorded zero chick-a-dee calls across all groups, phases, and playback types. Our full model could not achieve reliable convergence. Thus, we adopted a simpler model without interaction terms and playback phases to compare means and variances across stimulus playback types. Birds tended to produce more chick-a-dee calls during sequential playback types compared to solo playback types (evidence ratio=7.51, posterior probability of increase=88%), but with wide uncertainties (posterior mean=3.05, 95% CrI: 0.61-13.88; Fig. S3; Table S3). Birds produced different numbers of chick-a-dee calls across playback types, with WV eliciting the highest response [mean=2.3, 95% highest posterior density (HPD): 0.97-3.95], followed by VW (mean=0.85, 95% HPD: 0.41-1.39), VO (mean=0.67, 95% HPD: 0.25-1.17), and WO showing the lowest response (mean=0.3, 95% HPD: 0.06-0.66; Fig. 4). Birds produced more chick-a-dee calls during WV (evidence ratio=111.67, posterior probability of increase=99.1%) as compared to other presentations (VO, WO, and VW).

## DISCUSSION

Here, we examined how black-capped chickadees respond to predator and non-predator acoustic cues (i.e. wingbeats and calls) presented alone and sequentially. We did not find evidence that birds in predator and non-predator groups respond differently to predator and non-predator wingbeats and calls. Contrary to our expectations, birds did not respond more to predator wingbeats or calls. Generally, we found no evidence that the birds produced different responses to sequential playbacks as compared to solo playbacks. The variance or variability of response was lower (but had wider credible intervals) for sequential playbacks than solo playbacks. Chickadees produce more chick-a-dee calls in response to playbacks of predator calls and mobbing calls (Billings et al., 2015; Congdon et al., 2016; Templeton et al., 2005). In our study, the majority of birds did not produce any chick-a-dee calls in response to either predator or non-predator playback, but preliminary results suggest birds tended to produce more chick-a-dee calls during wingbeat-vocalization sequence as compared to other playback types. The anti-predator response to predator calls could be individual specific, and we would require a large sample size to comment on the pattern of response at the population level. The predator group birds produced lower numbers of perch hops and similar numbers tseet calls than non-predator group birds, but both groups had wider overlapping credible intervals. During the sequential playbacks (vocalization-wingbeat and wingbeat-vocalization), we did not find evidence that birds produced different numbers of perch hops and tseet calls than during solo playbacks (wingbeat and vocalization), which could be because of small sample size of the study. However, birds had lower variance in response during the sequential playbacks as compared to the solo playbacks. The observed behaviour during sequential playbacks could be due to arousal and/or sensitization to the stimuli as sequential playbacks were always after the solo playbacks in our experimental design. Most of the playback conditions had wide credible intervals suggesting low reliability of acoustic cues and/or individual specific response to the acoustic cues.

How would prey animals integrate information from the same sensory modality? The response following the integration of cues would depend on the novelty of the cue, context, and experience of the individual (Crane et al., 2024). Generally, within acoustic cues, mobbing calls may provide more certainty, predator calls moderate certainty, and wingbeats low certainty about the current predation risk (Amorim and Dias, 2019; Mathot et al., 2024). Perceptually, wingbeats of a species can be harder for birds to discriminate because of potentially incomplete or unreliable information (Crane et al., 2024). Wingbeats associated with predator calls may provide better assessment of the current threat. Our results suggest that sequential playbacks, wingbeat played before or after vocalization, had lower overdispersion as compared to the solo vocalization and wingbeat playbacks, which had very high overdispersion (transformed dispersion parameter closer to 0). This high variability in responses to single-modality cues suggests that individual birds may interpret these acoustic signals differently, possibly reflecting variation in prior experience. The reduced overdispersion with wingbeat-vocalization combination playbacks indicates that sequential acoustic information may provide a more reliable threat assessment framework. This temporal integration within the acoustic modality may represent an adaptive strategy for improving threat discrimination accuracy while minimizing false alarms. The inconsistent responses to the acoustic cues highlight the importance of considering individual variation and context-dependent factors when interpreting anti-predator behaviours in laboratory settings.

Our findings align with growing evidence that non-vocal cues including wingbeats can be crucial for threat assessment. Lima and Dill (1990) emphasized that behavioural decisions under predation risk involve complex trade-offs and uncertainty, suggesting that ambiguity in threat assessment may be more common than previously recognized in predator-prey interactions. Studies on non-vocal cues have shown that wing-generated sounds serve important signalling functions in many bird species (Clark and Areta, 2024; Clark and Prum, 2015), but their roles in predator threat detection are not well known. In crested pigeons (*Ocyphaps lophotes*), wing ‘whistles’ from conspecific individuals can function as alarm calls (Hingee and Magrath, 2009), and conspecific wing trill playbacks can elicit anti-predator responses in scaled doves (*Columbina squammata*) (Amorim and Dias, 2021), suggesting importance of conspecific non-vocal cues. However, Shearer and Beilke (2023) demonstrated that sparrows (Passerellidae) can recognize and respond to isolated wingbeat sounds (both predator and non-predator), suggesting that some species may have evolved the capacity for wingbeat-based threat discrimination. Our results suggest that black-capped chickadees may develop recognition mechanisms for wingbeats only when these cues are paired with more reliable acoustic information (e.g. calls), rather than being able to discriminate between predator and non-predator wingbeats in isolation. This lack of discrimination may result from our specific choice of predator and non-predator species, combined with the inherent variability in wingbeat characteristics that exist across different bird species. The reduced overdispersion to sequential acoustic cue presentations in our study suggests that temporal context may be important for effective threat assessment when dealing with uncertain acoustic cues.

Our results provide partial support for MLE integration theory in acoustic threat assessment. The reduced variance observed in sequential playbacks compared to solo playbacks aligns with MLE predictions that combining multiple cues should decrease response variability by providing more reliable threat information (Ernst and Banks, 2002; Mathot et al., 2024). However, we did not find evidence for differential responses to predator and non-predator wingbeats and vocalizations suggesting that black-capped chickadees may not be extracting reliable threat-level information from these acoustic cues. This pattern indicates that wingbeats may function more as alertness cues rather than specific threat-level indicators in this species. The reduced overdispersion to sequential presentations may therefore reflect an additive effect of acoustic cues than threat assessment. Studies with multiple acoustic cues (e.g. mobbing call, high-threat predator call, more samples of wingbeats) of various levels of predation risks are required to comment on black-capped chickadees' general threat assessment ability.

The current exploratory study had several limitations which should be considered when interpreting results and could be helpful for designing future studies. First, our sample size may have limited our ability to detect subtle differences in responses between predator and non-predator stimuli, particularly given the high individual variation observed in birds' responses. Second, the acoustic stimuli themselves carried inherent uncertainty; wingbeats and vocalizations were recorded from a limited number of individuals and species, potentially not representing the category of threat versus non-threat, and our use of only one type of wingbeat stimulus per species may have oversimplified the acoustic complexity of predator encounters. Third, behavioural measurement limitations prevented us from quantifying specific anti-predator behaviours, as we recorded general activity responses and relied on vocal responses. But the vocal response data (e.g. number of chick-a-dee call, high ‘zees’, gargles, ‘fee-bee’ song) for most of the birds were over dispersed thus we could not conduct any meaningful analyses. Fourth, we did not have control groups for different loudness for acoustic stimuli; thus the birds' responses partially may be due to the loudness of stimuli rather than the biological relevance, which limits the generalization of our results to more ecological settings. Future research can quantify anti-predator behaviours in larger experimental aviaries that allow for more natural movement patterns.

Overall, we did find evidence that black-capped chickadees respond differentially to both wingbeat and vocal cues from potential predators and non-predators. The reduced overdispersion to sequential acoustic cue playbacks suggest birds may integrate temporal acoustic information to make anti-predator decisions. Though we cannot isolate the interaction of playback order and playback type from our limited analysis. In the context of threat assessment, visual cues play a dominant role (Arteaga-Torres et al., 2020; Caro, 2005; Crane et al., 2024) and acoustic cues alone can also elicit behavioural response. The ability to respond to ambiguous acoustic cues through temporal integration suggests that acoustic environments could shape prey-predator interaction.

## MATERIALS AND METHODS

### Subjects

Sixteen (eight males and eight females) black-capped chickadees were used for the experiment between October 2023 and March 2025. Birds were captured between 2020 and 2024 from the North Saskatchewan River Valley (53.53°N, 113.53°W) and Mill Creek Ravine (53.52°N, 113.47°W) in Edmonton, Alberta, Canada and were determined to be adults through analysis of the tail rectrices (Pyle et al., 1997). Sex was determined using DNA analysis of blood samples (Griffiths et al., 1998). Prior to the experiment, birds were housed in individual cages (51×51×67 cm, King's Cages, East Brunswick, New Jersey, USA) within a colony room where they had visual and auditory contact with other birds. Birds had *ad libitum* access to food (Mazuri Insectivore Diet; Mazuri, St. Louis, MO, USA), water, grit (Rolf C. Hagen Inc., Montreal, Quebec, Canada), and cuttlebone. Five to eight sunflower seeds were also provided daily, as well as one mealworm dusted with vitamins (Prime Vitamin Supplement; Hagen Inc., Montreal, QB, Canada) three times per week, and hard-boiled eggs mixed with greens (parsley or spinach) twice per week. On experiment days (except during the playbacks), birds had *ad libitum* access to food, water, grit, and cuttlebone, as well as two mealworms per day. Birds were maintained on a light:dark cycle following the natural light cycle for Edmonton, AB, Canada.

### Apparatus

Throughout the experiment, birds were housed individually in a cage (30×40×40 cm, Rolf C. Hagen, Inc., Montreal, Quebec, Canada) inside a sound-attenuating chamber (inner dimensions 58×168×83 cm; Industrial Acoustics Corporation, Bronx, New York, USA). The cage was outfitted with a water bottle, food cup, and three equally spaced plastic perches. The food cup and water bottle were removed before the experiment began. Birds were monitored daily by video.

In each sound chamber, stimuli were played through an amplifier (Cambridge Audio, Azur 640A Integrated Amplifier; London, UK) to a speaker (Fostex FE108 Σ full range speaker; Fostex Corp., Japan; frequency response range 80-18,000 Hz) using an mp3 player (Creative ZEN, Singapore). Amplitude was measured at the level of the perches from the centre position of the cage and playback amplitude was set to ∼70-75 dB with a Sper Scientific Sound Level Pen 840018 (Sper Scientific, Scottsdale, AZ, USA). We chose a louder playback for the acoustic stimuli, as observed in the study by Shearer and Beilke (2023), louder stimuli had higher behavioural response, and we did not have more control groups with different levels of loudness of acoustic stimuli. The playback loudness of wingbeat stimuli may be higher than the natural wingbeat sound which might limit our ability to extend our findings to naturalistic settings. Audio recordings of the birds were obtained using AKG C 1000S condenser microphones (frequency response: 50-20,000 Hz; AKG Acoustics, Vienna, Austria), and solid-state recorders (Marantz PMD661, D&M Professional, Itasca, IL, USA). Video recordings of the experiment were obtained using a Sony Handycam DCR-SX45 video camera (Sony Electronics Asia Pacific Pte Ltd., Tokyo, Japan, or Canon VIXIA HF R500, Canon Canada Inc., Mississauga, Ontario, Canada).

### Acoustic stimuli and experimental design

Chickadees have demonstrated the ability to distinguish vocalizations of high-threat versus low-threat predators (Congdon et al., 2016). Additionally, research has shown that wingbeat sounds can convey information about nearby threats (Hingee and Magrath, 2009; Shearer and Beilke, 2023). We conducted playback trials using recordings of sharp-shinned hawk (*Accipiter striatus*) vocalizations (Cruickshank, 2012a,b), dark-eyed junco (*Junco hyemalis*) vocalizations (Driver, 2022), sharp-shinned hawk wingbeats, and dark-eyed junco wingbeats. Both sharp-shinned hawk and dark-eyed junco are sympatric to black-capped chickadees. Wingbeat stimuli were obtained through personal communication with David J. Shearer and Elizabeth A. Beilke (David J. Shearer, Ball State University, USA, personal communication). The sharp-shinned hawk's wingbeat was recorded as the bird flew from one perch to another in the direction of the microphone (Sennheiser Model ME66) covering 10 m distance. The dark-eyed junco's wingbeat was recorded as the bird departed from a feeding platform with a distance 0.5 m from the microphone (Sennheiser Model ME66). Of the vocalizations, there were two calls from the sharp-shinned hawk, a high-threat predator species and two calls from the dark-eyed junco, a small non-predator species. There was one wing beat recording from the sharp-shinned hawk, and one wing beat recording from the dark-eyed junco. Each audio clip contained ∼15-20 s of stimulus followed by ∼45-40 s of silence (see the supplementary material for the spectrograms of the stimuli). The audio clip (60 s) was played for 5 min during playback.

We used a mixed factorial design in which condition (predator and non-predator) was assigned as a between-subjects factor, with birds randomly allocated to one of two groups (predator: *N*=8; non-predator: *N*=8) and playback type (WO, VO, WV, VW) as within-subjects factor. Within each group (predator and non-predator), each bird received all four playback types (WO, VO, WV, VW) within a single session. Presentation order was pseudo-counterbalanced across four sequences, with two birds completing each sequence per group (Table 1). Solo-stimulus presentations (WO and VO) were always administered prior to sequential presentations (WV and VW), as presenting combined stimuli first would confound first-exposure responses to individual components, which could limit interpreting the results.

#### Predator group

Birds were presented with four playback types: (1) predator VO, (2) predator WO, (3) predator VW, and (4) predator WV.

#### Non-predator group

Birds received corresponding non-predator stimuli: (1) non-predator VO, (2) non-predator WO, (3) non-predator VW, and (4) non-predator WV.

### Procedure

All birds were housed individually in the sound attenuating chambers and were allowed to acclimate for ∼18-24 h prior to the commencement of experiment. Playback experiments commenced the following day, with each bird beginning with the respective stimulus randomly assigned for experimental condition. The experiment lasted for ∼60 min per bird. For each playback type (e.g. WO), the 5 min prior to and following playback were recorded for baseline and post-playback comparisons. Each of the playback types were presented consecutively, with the baseline phase commencing immediately following the post-playback recording for the previous playback type (Fig. 1). For both predator and non-predator groups each bird was exposed to all playback type sequences within 1 day in pseudo-randomized order, starting with their respective sequence (see Table 1). All the birds were tested in the morning with trials beginning between 09:15 and 09:30 each day.

### Response measures

To measure the effect of the presented stimuli, we observed birds' antipredator responses in the form of vocalizations and perch hopping. During each condition, we recorded the frequency and type of vocalizations, namely whether they exhibited chick-a-dee calls, tseet calls, high zees, gargle calls, and fee-bee songs. We focused our analysis on tseet and chick-a-dee calls as the majority of birds did not produce other vocalizations. Movement behaviour was observed by recording the number of perch hops performed as a proxy for activity (Congdon et al., 2016). K.N. and J.R. (blind to groups and playback types) extracted the responses with help of P.K.S. Through we did not perform formal inter-rater reliability test, lead observer (P.K.S.) cross-checked a subset of audio and video recordings for call and perch hop counts, which had high agreement among P.K.S., K.N., and J.R. Another variable time spent near the speaker was excluded from analysis because of poor reliability.

### Statistical analyses

All statistical analyses were conducted in R Statistical Software v 4.5.1 (R Core Team, 2024) with Bayesian mixed-effects models implemented in the brms package (Bürkner, 2017). We fitted separate models for each response variable: perch hops, tseet calls, and chick-a-dee calls. For perch hops and tseet calls, we used negative binomial mixed-effects models to account for overdispersion in count data. For chick-a-dee calls, we employed a Bayesian negative binomial hurdle model due to the high frequency of zero observations (∼70% of responses).

The general model structure included experimental groups (predator vs non-predator), phase (baseline, playback, post-playback), playback type (VO, WO, VW, WV), and their interaction as fixed effects, with individual bird ID as grouping factor with both a random intercept and a random slope for phase [e.g. perch hops∼perch_hops∼condition×type×phase+(1+phase|ID)]. The hurdle model for chick-a-dee calls included playback type effects in both the hurdle (zero-inflation) and count components. Our full hurdle model could not achieve reliable convergence. Thus, we adopted a simpler model without interaction terms and playback phases to compare means and variances across stimulus playback types [chickadee_call ∼condition+type+(1+ID), hu∼type+(1+ID)]. We used weakly informative priors based on the response variable and ran four MCMC chains with 4000 iterations each (2000 warmup) with adapt_delta set to 0.95 to improve sampling efficiency. We used weakly informative priors; [normal(0, 1)] on log scale for fixed effects and interaction coefficients and normal(0, 6) for the intercept, student_t(3,0,3) for variance fixed effect. Model convergence was assessed using R̂ values and effective sample sizes. We examined model fit using posterior predictive checks.

Hypothesis testing was conducted using directional Bayesian hypotheses with evidence ratios. Evidence ratio computes posterior probability under the hypothesis against its alternative (Bürkner, 2017). We tested specific contrasts including (1) predator versus non-predator effects, (2) sequential versus solo playback type effects, and (3) phase-specific responses. Results are reported as posterior means with 95% credible intervals, along with evidence for directional hypotheses. We also examined the dispersion parameter across playback types to assess response variability.

Generally, Bayesian models yield a posterior distribution, which is a probability distribution over parameter values (e.g. mean, median) that incorporates both the data and the prior. To quantify evidence for directional hypotheses (e.g. predator condition elicits more tseet calls than non-predator condition), we report the posterior probability that a parameter exceeds zero (or some threshold of interest), computed as the proportion of posterior draws satisfying that inequality. Higher values can be treated as strong directional evidence. Where we test whether a parameter differs in groups and conditions, we report evidence ratios: the ratio of posterior probability mass on one side of a hypothesis to the other (e.g. an evidence ratio of 19 can be considered as ‘strong’, corresponding to a posterior probability of ∼0.95). Unlike null-hypothesis significance testing, these quantities do not require a binary accept/reject decision and allow graded interpretation of evidence strength.

### Ethical note

This research was conducted in accordance with the Canadian Council on Animal Care (CCAC) Guidelines and approved from the Animal Care and Use Committee for Biosciences for the University of Alberta, Canada. All birds capture and research were authorized through appropriate permits: Canadian Wildlife Service Scientific permits [13-AB-SC004_(2019,2020,2021,2022)_, |SCPR-2023-AB-013A _(2023)_], Province of Alberta Capture and Research permits [#19–020_(2019)_, #20–084_(2020)_, #21–098_(2021)_, #22–017_(2022)_, #23015_(2023)_] and a North Saskatchewan River Valley Area Redevelopment Plan (NSRV ARP) permit.

## Supplementary Material



10.1242/biolopen.062598_sup1 Supplementary information
