# Supplementary material for: Differential response variability of black-capped chickadees to wingbeat sounds and vocalizations
Source: Biol Open. 2026 Jun 24;15(6):bio062598. doi: 10.1242/bio.062598 (PMC13382834; doi:10.1242/bio.062598)
Supplement: Supplementary information [file biolopen-15-062598-s1.pdf]

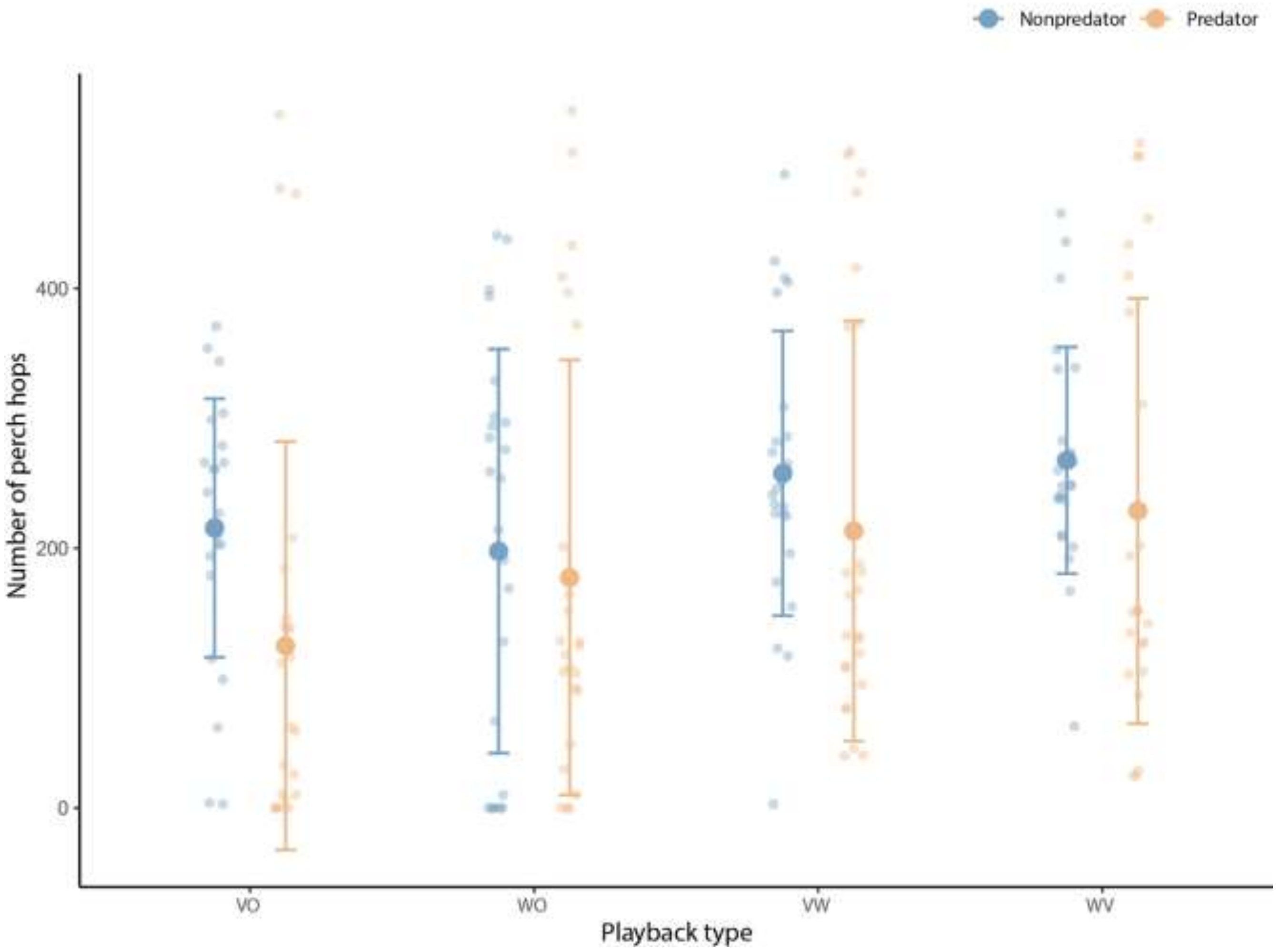

Fig. S1. Number of perch hops by playback type and experimental condition. Filled circles represent mean and error bars represent standard deviation with jitter showing each data point (N=8 for each non-predator and predator). Playback types: VO = vocalization only, WO = wingbeat only, VW = sequential vocalization-wingbeat, WV = sequential wingbeat-vocalization.

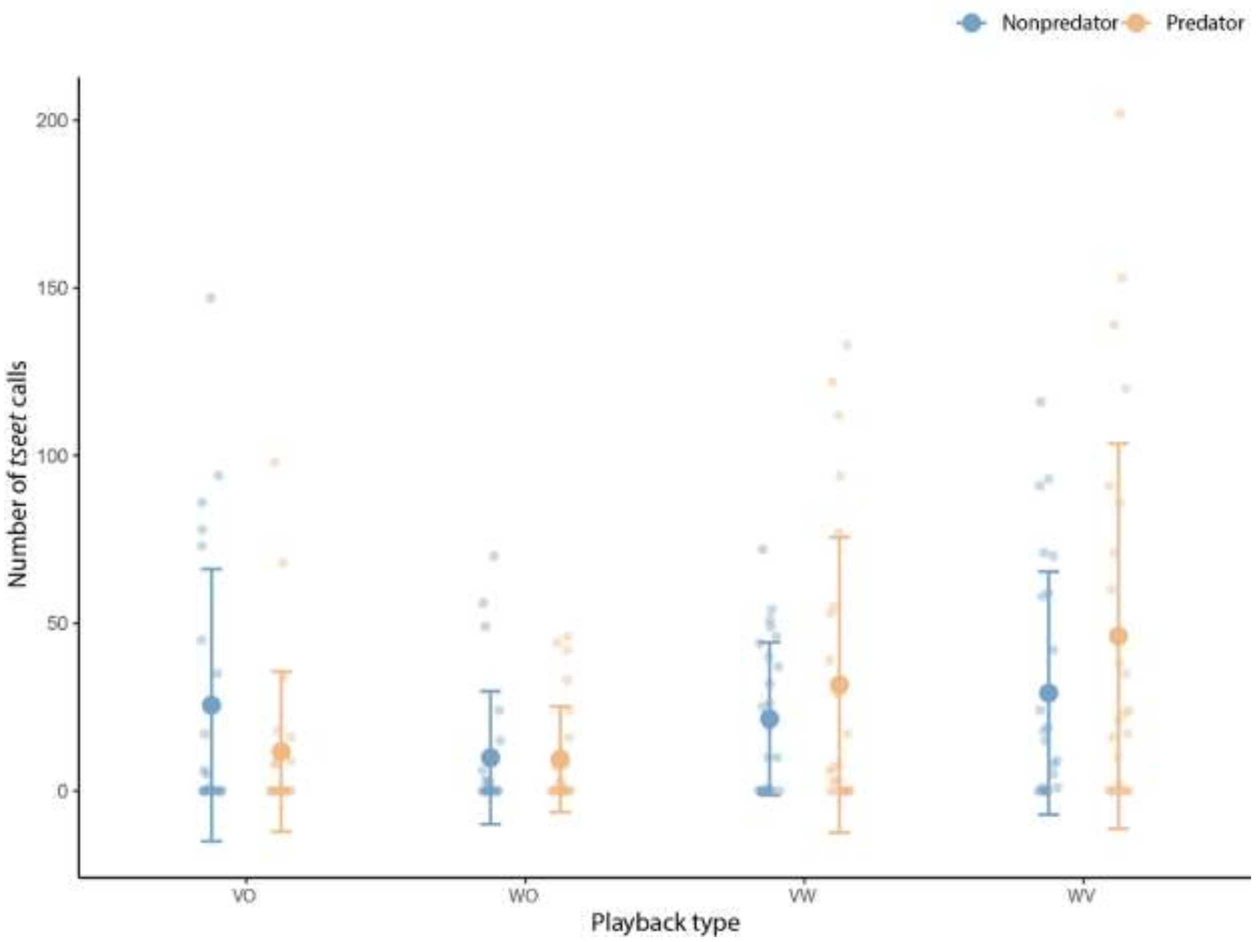

Fig. S2. Number of *tseet* calls by playback type and experimental condition. Filled circles represent mean and error bars represent standard deviation with jitter showing each data point (N=8 for each non-predator and predator). Playback types: VO = vocalization only, WO = wingbeat only, VW = sequential vocalization-wingbeat, WV = sequential wingbeat-vocalization.

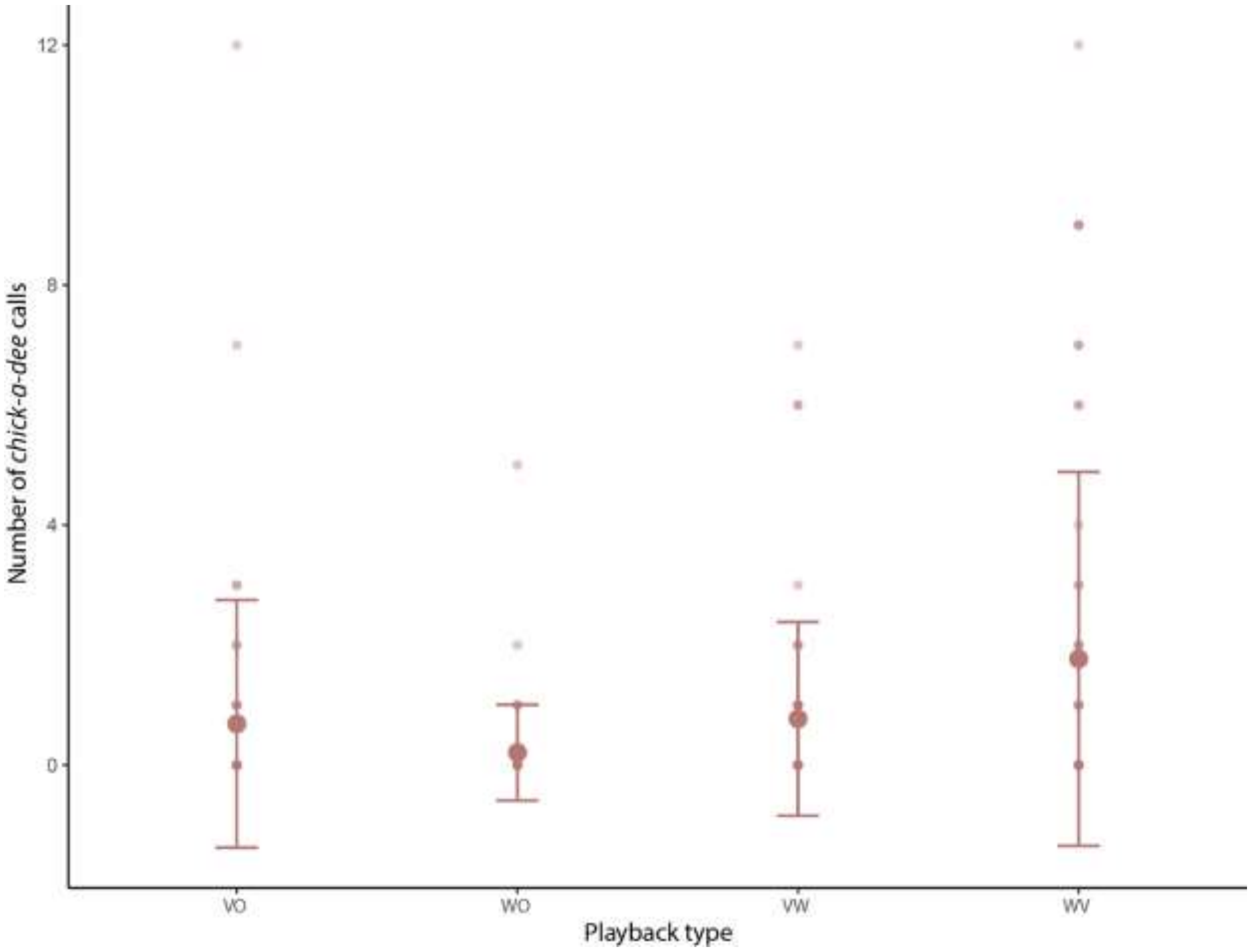

Fig. S3. Number of *chick-a-dee* calls by playback type. Filled circles represent mean and error bars represent standard deviation with jitter showing each data point (N=16 for each playback type). Playback types: VO = vocalization only, WO = wingbeat only, VW = sequential vocalization-wingbeat, WV = sequential wingbeat-vocalization.

Biology Open • Supplementary information

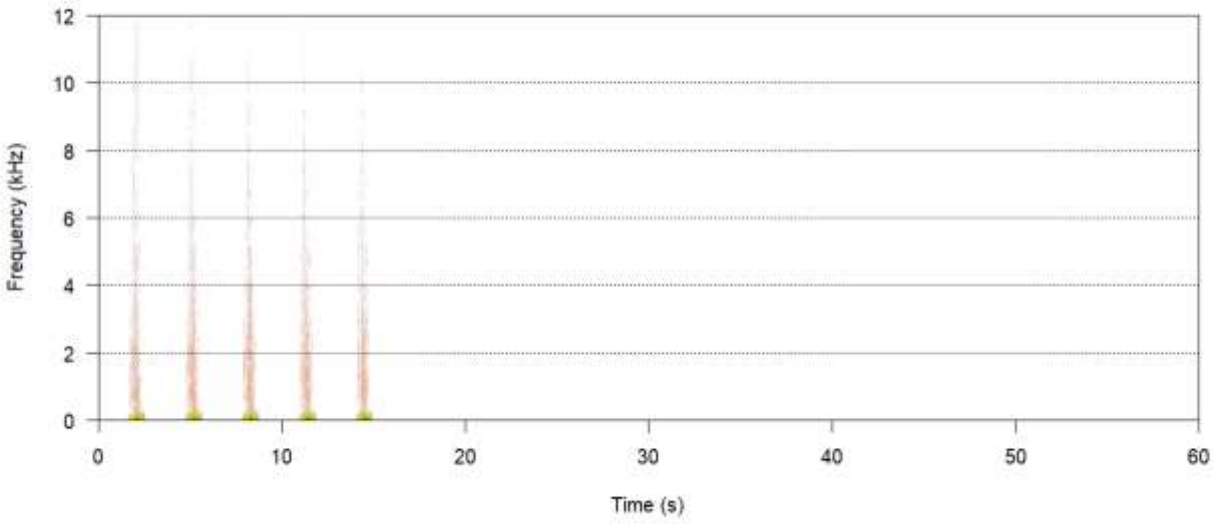

Fig. S4. Spectrogram of Sharp-shinned hawk wingbeat acoustic stimuli

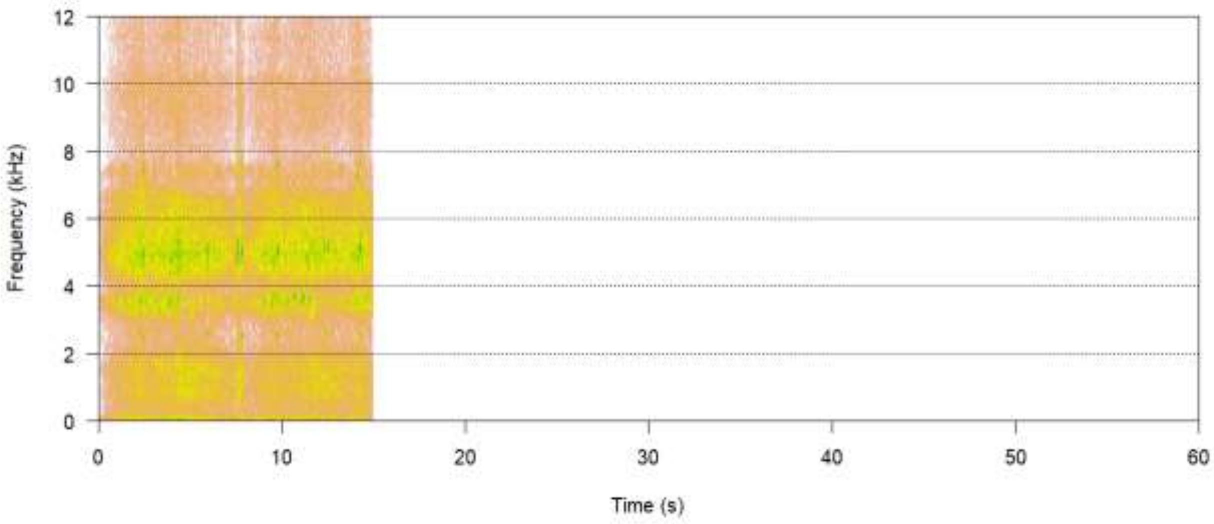

Fig. S5. Spectrogram of Sharp-shinned hawk vocalization acoustic stimuli

Biology Open • Supplementary information

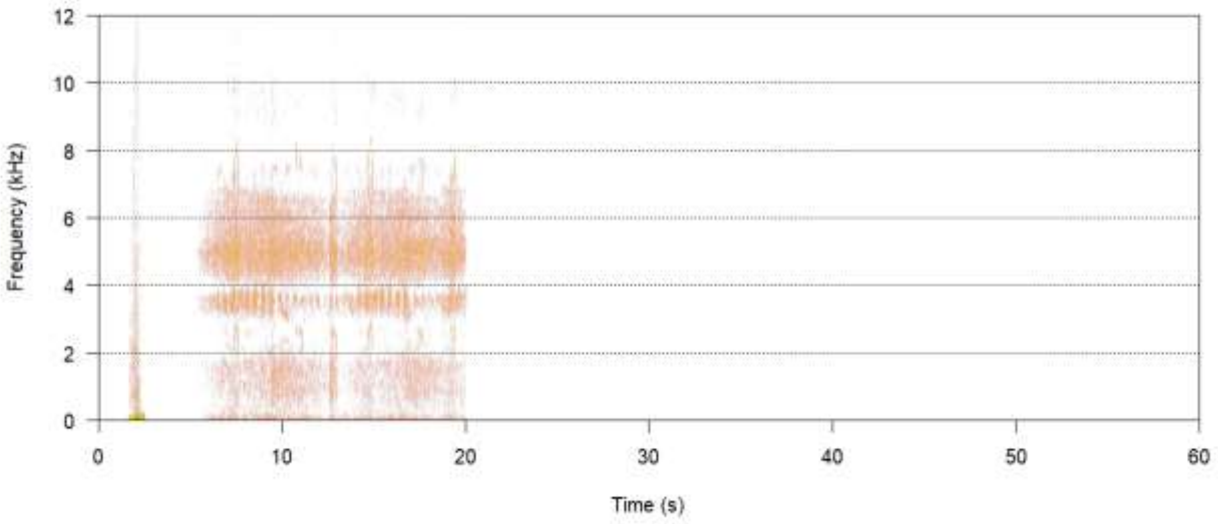

Fig. S6. Spectrogram of Sharp-shinned hawk wingbeat-vocalization acoustic stimuli

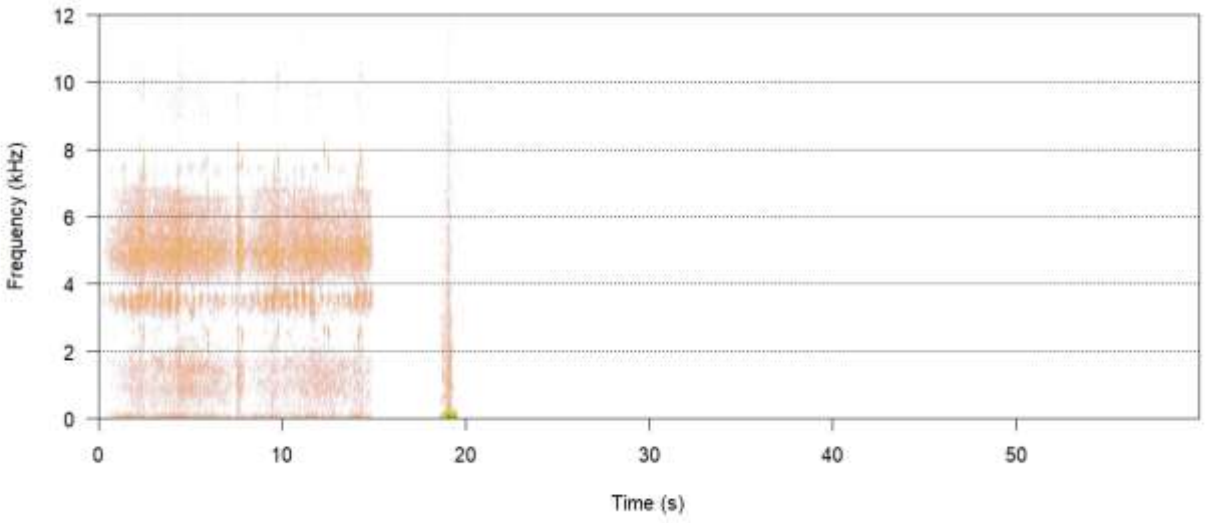

Fig. S7. Spectrogram of Sharp-shinned hawk vocalization-wingbeat acoustic stimuli

Biology Open • Supplementary information

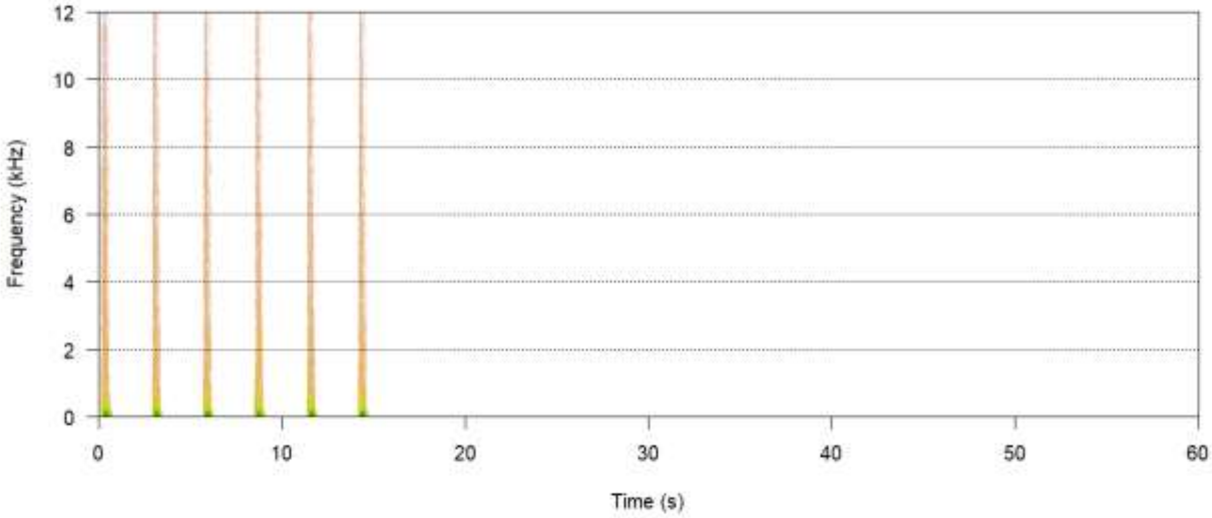

Fig. S8. Spectrogram of Dark-eyed Junco wingbeat acoustic stimuli

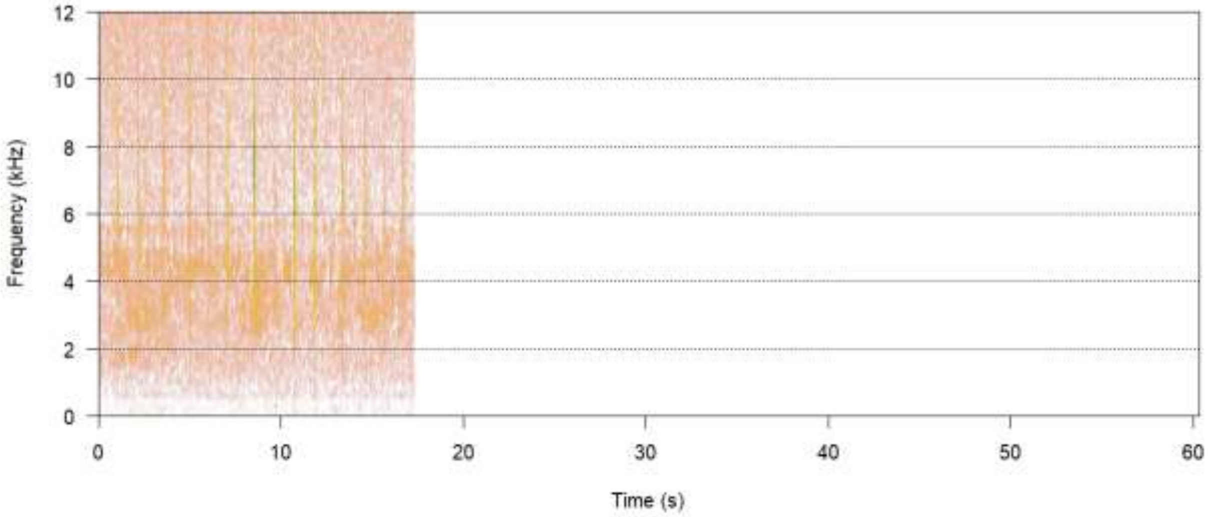

Fig. S9. Spectrogram of Dark-eyed Junco vocalization acoustic stimuli

Biology Open • Supplementary information

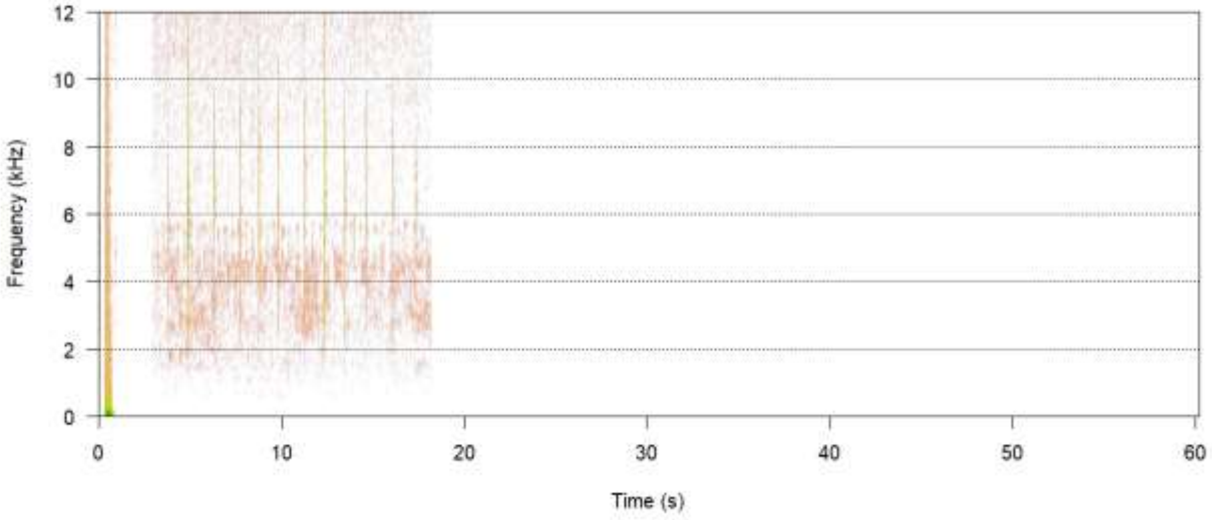

Fig. S10. Spectrogram of Dark-eyed Junco wingbeat-vocalization acoustic stimuli

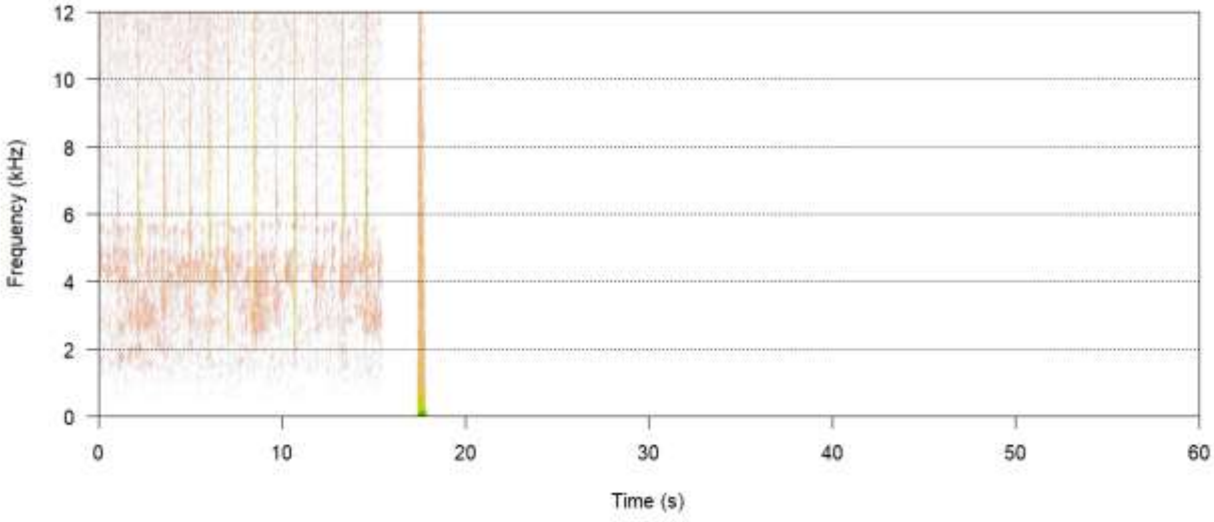

Fig. S11. Spectrogram of Dark-eyed Junco vocalization-wingbeat acoustic stimuli

Table S1. Model output of Activity response (Perch hops) analysis. Family: negbinomial. Links: mu = log; shape = log. Formula: perch_hops ~ condition * type * phase + (1 + phase | ID)

	Estimate	Est.Error	l-95% CI	u-95% CI	Rhat	Bulk_ESS	Tail_ESS
Intercept	5.42	0.35	4.73	6.13	1.00	2503	3831
shape_Intercept	-0.42	0.20	-0.82	-0.05	1.00	8993	6784
conditionPredator	-0.56	0.45	-1.44	0.31	1.00	3070	4403
typeVW	0.14	0.29	-0.44	0.70	1.00	4001	5362
typeWO	0.02	0.41	-0.77	0.83	1.00	5522	6161
typeWV	0.13	0.27	-0.41	0.66	1.00	3710	5195
phasePlayback	-0.11	0.35	-0.81	0.58	1.00	3065	4674
phasePostplayback	-0.12	0.35	-0.81	0.57	1.00	3655	5244
conditionPredator:typeVW	0.13	0.37	-0.62	0.85	1.00	4770	5327
conditionPredator:typeWO	0.11	0.52	-0.92	1.14	1.00	7024	6175
conditionPredator:typeWV	0.07	0.34	-0.60	0.72	1.00	4387	5512
conditionPredator:phasePlayback	0.13	0.44	-0.73	1.00	1.00	3831	5218
conditionPredator:phasePostplayback	0.01	0.43	-0.85	0.87	1.00	4538	5380
typeVW:phasePlayback	0.07	0.39	-0.67	0.83	1.00	3512	5094
typeWO:phasePlayback	-0.17	0.55	-1.27	0.93	1.00	6376	5981
typeWV:phasePlayback	0.08	0.36	-0.63	0.78	1.00	3193	4951
typeVW:phasePostplayback	-0.12	0.38	-0.85	0.61	1.00	3777	5333
typeWO:phasePostplayback	-0.06	0.56	-1.14	1.05	1.00	6796	5955
typeWV:phasePostplayback	0.02	0.35	-0.67	0.70	1.00	3654	5111
conditionPredator:typeVW:phasePlayback	0.09	0.48	-0.83	1.01	1.00	4092	5496
conditionPredator:typeWO:phasePlayback	0.04	0.68	-1.31	1.37	1.00	8982	5733
conditionPredator:typeWV:phasePlayback	0.08	0.44	-0.79	0.94	1.00	3898	5446
conditionPredator:typeVW:phasePostplayback	0.12	0.48	-0.83	1.05	1.00	5017	5909
conditionPredator:typeWO:phasePostplayback	0.03	0.69	-1.33	1.38	1.00	8508	6751

Biology Open • Supplementary information

conditionPredator:typeWV:phasePostplayback	0.21	0.43	-0.64	1.06	1.00	4625	5583
shape_typeVW	2.14	0.32	1.51	2.75	1.00	8472	5750
shape_typeWO	-0.30	0.29	-0.86	0.27	1.00	10437	6954
shape_typeWV	4.32	1.25	3.08	7.52	1.00	548	424

Table S2. Model output of Vocal activity (Tseet calls) analysis. Family: negbinomial. Links: mu = log; shape = log. Formula: tseet ~ condition * type * phase + (1 + phase | ID) shape ~ type

	**Estimate**	**Est.Error**	**l-95% CI**	**u-95% CI**	**Rhat**	**Bulk_ESS**	**Tail_ESS**
Intercept	1.24	0.98	-0.74	3.11	1.00	1560	3211
shape_Intercept	-1.07	0.33	-1.71	-0.40	1.00	7252	6272
conditionPredator	-0.09	0.83	-1.71	1.56	1.00	3763	5378
typeVW	0.40	0.46	-0.49	1.29	1.00	5927	6694
typeWO	0.16	0.56	-0.93	1.28	1.00	9010	6546
typeWV	0.14	0.48	-0.81	1.08	1.00	6834	5898
phasePlayback	-0.37	0.52	-1.41	0.63	1.00	6916	6497
phasePostplayback	0.46	0.50	-0.50	1.46	1.00	5296	6102
conditionPredator:typeVW	0.52	0.54	-0.56	1.62	1.00	8238	6205
conditionPredator:typeWO	-0.11	0.68	-1.44	1.24	1.00	8228	5899
conditionPredator:typeWV	1.00	0.59	-0.17	2.17	1.00	7941	6677
conditionPredator:phasePlayback	0.38	0.60	-0.79	1.55	1.00	6870	6293
conditionPredator:phasePostplayback	-0.40	0.62	-1.60	0.81	1.00	4683	4904
typeVW:phasePlayback	-0.39	0.56	-1.46	0.72	1.00	7277	6585
typeWO:phasePlayback	-0.16	0.71	-1.52	1.29	1.00	10158	6447
typeWV:phasePlayback	0.36	0.60	-0.82	1.57	1.00	7775	6241
typeVW:phasePostplayback	-0.14	0.58	-1.30	1.01	1.00	5929	6002
typeWO:phasePostplayback	-0.52	0.73	-1.94	0.98	1.00	8746	5771
typeWV:phasePostplayback	0.46	0.61	-0.72	1.64	1.00	4810	4754
conditionPredator:typeVW:phasePlayback	-0.46	0.69	-1.78	0.90	1.00	7547	5708
conditionPredator:typeWO:phasePlayback	-0.16	0.84	-1.81	1.46	1.00	11779	6133
conditionPredator:typeWV:phasePlayback	0.95	0.72	-0.48	2.37	1.00	9490	6756
conditionPredator:typeVW:phasePostplayback	0.11	0.69	-1.19	1.44	1.00	6852	6435
conditionPredator:typeWO:phasePostplayback	-0.02	0.83	-1.68	1.61	1.00	10802	6205
conditionPredator:typeWV:phasePostplayback	0.75	0.73	-0.69	2.15	1.00	7954	6297
shape_typeVW	1.23	0.75	0.04	2.97	1.00	1062	457
shape_typeWO	-0.42	0.47	-1.37	0.48	1.00	7348	6488
shape_typeWV	0.66	0.43	-0.18	1.50	1.00	6239	5676

Biology Open • Supplementary information

Table S3. Model output of Vocal activity (Chick-a-dee calls). Family: hurdle_negbinomial Links: mu = log; shape = identity; hu = logit. Formula: chickadee_call ~ condition + type + (1 + ID) hu ~ type + (1 + ID)

	Estimate	Est.Error	l-95% CI	u-95% CI	Rhat	Bulk_ESS	Tail_ESS
Intercept	-1.52	3.03	-10.57	1.01	1.00	1469	1751
hu_Intercept	0.93	0.62	-0.27	2.15	1.00	2898	4676
conditionPredator	0.05	0.60	-1.17	1.22	1.00	5615	6020
typeVW	-0.29	0.55	-1.48	0.74	1.00	5679	4995
typeWO	-0.41	0.69	-1.75	0.96	1.00	8261	5361
typeWV	1.00	0.53	-0.04	2.09	1.00	6578	6175
IDG56	-0.69	0.81	-2.30	0.94	1.00	7502	5552
IDG603	-0.74	0.76	-2.21	0.83	1.00	8486	5486
IDG613	-0.12	0.72	-1.48	1.37	1.00	8576	5384
IDG624	-0.10	0.85	-1.73	1.63	1.00	9585	5758
IDG628	-0.38	0.79	-1.91	1.19	1.00	8341	5214
IDG629	0.00	0.99	-1.96	1.95	1.00	8171	5299
IDG670	-0.15	0.76	-1.62	1.41	1.00	8846	5616
IDG671	-0.29	0.75	-1.77	1.21	1.00	8106	5546
IDG673	1.39	0.62	0.14	2.56	1.00	4635	5203
IDG679	-0.00	1.00	-1.95	1.92	1.00	10353	5636
IDG688	0.94	0.59	-0.21	2.07	1.00	5686	5224
IDG717	-0.00	1.01	-2.00	1.99	1.00	8867	5906
IDG719	-0.26	0.68	-1.60	1.10	1.00	7841	6043
IDG732	0.50	0.66	-0.81	1.84	1.00	7958	5285
IDS60	-0.00	0.99	-1.92	1.91	1.00	10155	5777
hu_typeVW	-0.84	0.54	-1.91	0.19	1.00	5637	5117
hu_typeWO	1.04	0.66	-0.20	2.38	1.00	6060	5431
hu_typeWV	-1.10	0.53	-2.14	-0.07	1.00	6303	5884
hu_IDG56	0.62	0.87	-1.03	2.41	1.00	4634	5208
hu_IDG603	0.16	0.82	-1.41	1.81	1.00	4206	5383
hu_IDG613	0.63	0.85	-1.00	2.38	1.00	4426	5157
hu_IDG624	2.05	1.17	-0.00	4.69	1.00	5234	4382
hu_IDG628	0.62	0.86	-1.01	2.36	1.00	4482	5246
hu_IDG629	4.66	2.94	1.01	12.32	1.00	3967	2373
hu_IDG670	0.64	0.86	-0.99	2.41	1.00	4403	5310
hu_IDG671	0.16	0.81	-1.42	1.81	1.00	3929	4770
hu_IDG673	-0.26	0.80	-1.83	1.33	1.00	3860	4302
hu_IDG679	4.73	3.17	1.00	12.99	1.00	3915	2227
hu_IDG688	-1.99	0.89	-3.81	-0.35	1.00	4443	4833
hu_IDG717	4.69	2.91	1.03	12.28	1.00	4617	2666
hu_IDG719	-0.26	0.80	-1.85	1.34	1.00	4152	5435
hu_IDG732	0.63	0.85	-1.05	2.36	1.00	4135	4865
hu_IDS60	4.82	3.24	0.99	13.40	1.00	3476	1962

	Estimate	Est.Error	l-95% CI	u-95% CI	Rhat	Bulk_ESS	Tail_ESS
shape	1.46	4.00	0.00	6.92	1.00	1181	1740

Biology Open • Supplementary information
