# Supplementary material for: Differential response variability of black-capped chickadees to wingbeat sounds and vocalizations
Source: Biol Open. 2026 Jun 24;15(6):bio062598. doi: 10.1242/bio.062598 (PMC13382834; doi:10.1242/bio.062598)
Supplement: Supplementary file 1 [file biolopen-15-062598_review_history.pdf]

Differential response variability of black-capped chickadees to wingbeat sounds and vocalizations

Prateek K. Sahu, Kailyn Nottebrock, Julian Ratch, Sarah Smeltz, Katharine Stenstrom, Moriah Deimeke and Christopher Sturdy
10.1242/bio.062598

Editor: Kendra J. Greenlee

Review timeline
Original submission: 31 March 2026
Editorial decision: 8 April 2026
First revision received: 2 June 2026
Accepted: 4 June 2026

Original submission

First decision letter

MS ID#: bio.062598

MS Title: Differential response variability of black-capped chickadees to wingbeat sounds and vocalizations

Authors: Prateek K. Sahu; Kailyn Nottebrock; Julian Ratch; Sarah Smeltz; Katharine Stenstrom; Moriah Deimeke; Christopher Sturdy

I have now reached a decision on the above manuscript.

The reviewer reports are shown at the bottom of this email.

As you will see, the reviewers raised a number of substantial criticisms that prevent me from accepting the paper at this stage. In particular, the reviewers expressed concerns about the statistical analysis, including some risk of overfitting due to low sample sizes. Scaling back the conclusions based on this would be beneficial. Addition of details on how to interpret the Bayesian statistics and figures showing raw data will improve the manuscript.

If you think that you can deal satisfactorily with the criticisms on revision, I would be pleased to see a revised manuscript.

At this stage, we also ask you to ensure your manuscript complies with our formatting guidelines. Provided you are able to fully address the referees' comments, we are positive about publication of your paper (we accept over 95% of revision submissions) and therefore hope you won't mind any extra work involved in reformatting your manuscript at this point.

Please upload both a 'clean' version of your Word file, along with a highlighted version clearly showing where you have made changes in the revised manuscript. Please avoid using 'Track changes' in Word files as these are lost in PDF conversion.

I should be grateful if you would also provide a point-by-point response detailing how you have dealt with the points raised by the reviewers in the 'Response to Reviewers' box. Please attend to all of the reviewers' comments. If you do not agree with any of their criticisms or suggestions please explain clearly why this is so.

© 2026. Published by The Company of Biologists under the terms of the Creative Commons Attribution License (https://creativecommons.org/licenses/by/4.0/).

Reviewer 1

Comments for the author

In this work, Sahu and colleagues investigate how black-capped chickadees respond to predator-derived sounds, whether these are vocalizations or the sound of wings in motion. They test if chickadees respond more strongly when the two types of sounds are combined in playback experiments than when exposed to either vocalizations or wingbeats alone. They also test for differences depending on whether the sounds come from a predator or a non-predator.

This is an interesting and compelling study, but several points would benefit from clarification, particularly regarding wingbeat stimuli used in the playbacks, and the model structure, including checks for overfitting and the specification of the random effects. Specifically:

* Where do the wingbeat sounds come from, and how far was the flying animal from the recording equipment?

* Even though the experiment was conducted in captivity, the setup should reflect naturalistic conditions. Decibel level itself could affect the response of the animals (e.g. louder than in nature?)

Abstract:

-It could be slightly restructured. Lines 30-31 seem somewhat disconnected and could be blended. The experimental design (predator vs non-predator playback) should be introduced before presenting the results.

-Line 41: It is unclear what "temporal" refers to.

Introduction:

- It would be helpful to specify in which species the cited evidence has been observed. It is not clear whether this refers only to birds or to other taxa as well.

- Is there any evidence that diurnal predators have evolved silent flight similar to some nocturnal birds? Do these predators typically approach prey in a glide, or is wingbeat noise common within a range where prey could hear it?

Methods:

- A brief note on the behavioral ecology of this species would be useful. Do they live in groups? How do they behave naturally in terms of predator avoidance? This could be mentioned in the introduction too.

- Line 163: What is meant by "Wingbeat stimuli were obtained through personal communication"?

- Line 167: Where were the wingbeat recordings obtained? Were the two wingbeat recordings matched in intensity? It is noted that wingbeat playbacks were presented at ~70-75 dB at perch level. Could the authors clarify whether this amplitude is biologically realistic for a predator's wingbeat as perceived by chickadees in nature? Playback at this intensity may be higher than naturally occurring wingbeat sounds and could elicit responses driven primarily by volume rather than the acoustic structure of the stimulus. A brief justification of the chosen amplitude or reference to natural wingbeat levels would strengthen confidence in the ecological validity of the results.

- If I understood correctly, the model includes a three-way interaction (condition × phase × playback type), resulting in 24 combinations with only 8 individuals per group. Could the authors

© 2026. Published by The Company of Biologists under the terms of the Creative Commons Attribution License (https://creativecommons.org/licenses/by/4.0/).

clarify how they assessed potential overfitting and ensured the robustness of these interaction estimates?

- Line 220: Specifying "bird ID nested within phase" may misrepresent the repeated-measures structure, treating the same individual in different phases as separate units. This can underestimate within-individual dependence and inflate the effective sample size. Modelling bird ID as a random intercept (and optionally random slopes) would better account for repeated observations.

Discussion

-Line 354: It would be helpful to clarify whether predator and non-predator stimuli contain the same proportion of sound and silence. This includes not only the silent intervals between sounds but also how the sounds are structured overall. Providing spectrograms in the supplementary materials would be useful for both vocalizations and wingbeat sounds.

-Additionally, could the authors comment on whether the non-predator wingbeat might be too similar to the wingbeats of chickadees themselves? This could influence how the birds perceive and respond to these stimuli.

Figures:

Figures 2 and 3 have panels of different sizes, one with a title and one without. It would improve clarity and consistency to make the panels homogeneous, so they do not appear as two independent figures placed side by side.

Reviewer 2

Comments for the author

This manuscript explores the role of acoustic cues in predator detection in black-capped chickadees (Poecile atricapillus), with a focus on the potential integration of wingbeat and vocalization signals. Using playback experiments, the authors examine whether chickadees discriminate between predator and non-predator cues and whether temporal integration of acoustic information influences their responses. The study addresses an interesting and relevant question in the context of animal communication. Overall, the work has the potential to contribute to our understanding of within-modality cue integration, although several aspects of the experimental design and interpretation would benefit from further clarification.

Main issues: I think the authors tend to interpret the lack of evidence to reject the alternative hypothesis (which could be due to several aspects, e.g. lack of power) as evidence of no difference in bird responses or that birds respond similarly. Also, I believe there are some interpretations that are not entirely supported by the statistics they have chosen. I think they should acknowledge this and draw conclusions with greater caution.

The paper presents the analyzed results using Bayesian statistics; therefore, the figures present predicted data. I think it will increase transparency if you include exploratory graph for raw data showing their natural dispersion as supplementary file. It would be great to have an equivalent figure to those of the main manuscript but showing true data spread.

L. 35-36 "…we found that chickadees did not respond more strongly to predator versus non-predator acoustic cues when presented individually". This result is an interpretation from data presented in Fig. 2 and 3. Credible intervals are wide, suggesting low precision. So, the lack of differences could be due to other reasons apart from birds not responding differently. I suggest being more cautious with the interpretation of the lack of differences and say something like: "…we found no differences in how chickadees responded to predator versus non-predator acoustic cues when presented individually".

© 2026. Published by The Company of Biologists under the terms of the Creative Commons Attribution License (https://creativecommons.org/licenses/by/4.0/).

L. 155 Could you add spectrograms of the stimuli you used? It would be useful for readers to know what range of frequencies these stimuli cover and what frequencies are predominant. It could be part of the supplementary data.

L. 174 Explain what WO, VO, WV, and VW represent. In the previous paragraph it is mentioned what VP, VN, WP, and WN are, but they are not used elsewhere after that. Then, the authors introduce these new abbreviations without describing them after the first use. It is a bit confusing for the reader.

L. 175 "each bird received all four playbacks" It is not clear what four playbacks you mean. It would be better for understanding the design to read the text in lines 181-186 first. For example, it could be placed right after mentioning WO, VO, WV and VW for the first time.

L. 177-180 Single-cue playbacks were always delivered before sequential playbacks. Therefore, the differences you find when you compare these groups might be due to arousal or sensitization. This should be more clearly stated in the discussion of the results. Although the authors mention this in the Methods section: "Single-stimulus presentations (WO and VO) were always administered prior to sequential presentations (WV and VW) … which could limit interpreting the results." this is not discussed elsewhere.

L. 189 It is not clear to me whether, during the 5-minute playback, the total duration of sound presented was the same regardless of whether it was a single-cue or sequential playback. In other words, if each audio lasted 1 minute, was the same audio played five consecutive times in the single-cue condition, while in the sequential condition one stimulus was played three times and the other twice (it is also unclear in which order)? Alternatively, was the single-cue condition structured as 1 minute of audio followed by 1 minute of silence, and so on, resulting in a total amount of sound that is half of that in the other condition? If the former is true, the differences among these stimuli (single vs. sequential) could be due to additive stimulation or increased acoustic exposure.

L. 199 Did you test more than one bird per day? If so, could the starting time any effect on the responses (e.g. it might be that birds are more responsive early in the morning than in the afternoon)?

L. 210 Have you performed an inter-rater reliability test among your observers?

L. 220-222 Did the hurdle model for the chick-a-dee calls only include playback type as an effect? Could you explain further why you did not include phase and bird ID, as in the other models?

L. 223 Could you specify which priors were used.

L. 227-233 I think it would be better to understand the results if you could elaborate on how to interpret Bayesian results. I know that Bayesian statistics are widely used in science, but in some disciplines, there are people not entirely with them yet. I believe it will improve the manuscript if you can provide a brief explanation of how to interpret evidence ratios, evidence for directional hypothesis, posterior probabilities, etc.

L. 231 Why did you use both means and medians along the manuscript? Could you explain why you chose one over the other?

L. 234 Could you provide the tables associated with the models you interpret? I wonder if it is appropriate to provide general data about predator vs non-predator groups when it is unclear whether interactions were significant or not.

L. 240-244 These results are a bit confusing. It states that birds did not respond differently across phases, yet it also says that playback and post-playback phases were 11% lower than baseline. If I understood correctly, the results show no differences among phases. It is ok to report the mean values, SD and range for each phase, but providing a magnitude of the difference when no difference is supported might lead to confusion. A similar situation occurs in L. 249-252, 285-288, and 293-294.

© 2026. Published by The Company of Biologists under the terms of the Creative Commons Attribution License (https://creativecommons.org/licenses/by/4.0/).

L. 245-252 Are you combining predator and non-predator groups responses? I believe you should not, because you do not expect the same magnitude of anti-predator responses for both groups; however, please make it clear in the text which group you are analyzing.

L. 249-251 "Birds responded with 30% more perch hops to combined playback types (VW and WV) compared to solo playback types (VO and WO) (evidence ratio = 2.37, posterior probability of increase = 70%)" Would not be more accurate to say that you found no differences between combined vs solo playback types, given that evidence ratio and probability?

L. 267-274 The interpretation of the results related to variability or dispersion in the responses seem somewhat ambiguous. It states that "Sequential combined playback types showed substantially higher dispersion parameter values than solo playback types, indicating less overdispersion response patterns during sequential presentations". I believe it might help to report the magnitude of these effects and provide back-transformed estimates would improve interpretability.

L. 325-327 I believe this result should be presented with more caution. With a posterior probability of 88%, it would be more appropriate to refer to a tendency. I understand that threefold difference is large, but (1) you are working with small numbers (as noted in L. 323 "Approximately 70% of observations recorded zero chick-a-dee calls across all conditions"); and (2) only one of the sequential playbacks supports that difference, the other sequential playback (VW) is similar to the solo vocalization playback (median 0.8 vs 0.62).

L. 330 Please include the meaning of HPD in the main text.

L. 350-352 Could you elaborate further on this preliminary analysis? I may have missed the part where you statistically compare wingbeat-vocalization sequence vs. the other conditions. Perhaps this could be included as a separate analysis in the supplementary material.

L. 369-370 "Our results suggest that wingbeat played before vocalization elicited higher response and had lower overdispersion." I do not think it is appropriate to draw this conclusion if the WV playback was not analyzed independently.

L. 371 "range included negative values" Could you back-transform the coefficients to aid interpretation?

Reviewer's Responses to Questions

Experimental quality
Does each figure have the proper controls?
If 'No', please indicate reasons in Comments for Author box below.
Reviewer #1:

- Yes

Reviewer #2:

- Yes

Were the data analyzed using appropriate statistical tests?
If 'No', please indicate reasons in Comments for Author box below.
Reviewer #1:

- Yes

Reviewer #2:

- Yes

© 2026. Published by The Company of Biologists under the terms of the Creative Commons Attribution License (https://creativecommons.org/licenses/by/4.0/).

Reproducibility
Were experiments performed using adequate number of biological replicates?
If 'No', please indicate reasons in Comments for Author box below.
Reviewer #1:

- No

Reviewer #2:

- Yes

Does the methods section provide sufficient detail to permit reproducibility?
If 'No', please indicate reasons in Comments for Author box below.
Reviewer #1:

- No

Reviewer #2:

- No

Completeness
Are the manuscript's conclusions supported by the data?
If 'No', please indicate reasons in Comments for Author box below.
Reviewer #1:

- Yes

Reviewer #2:

- No

Scholarship
Do the authors cite and discuss the merits of data that would argue for and against their conclusion?
If 'No', please indicate reasons in Comments for Author box below.
Reviewer #1:

- Yes

Reviewer #2:

- Yes

Does the manuscript title & abstract accurately reflect the contents of the manuscript, without hyperbole?
If 'No', please indicate reasons in Comments for Author box below.
Reviewer #1:

- Yes

Reviewer #2:

- Yes

First revision

Author response to reviewers' comments

Reviewer 1: In this work, Sahu and colleagues investigate how black-capped chickadees respond to predator-derived sounds, whether these are vocalizations or the sound of wings in motion. They test if chickadees respond more strongly when the two types of sounds are combined in playback

© 2026. Published by The Company of Biologists under the terms of the Creative Commons Attribution License (https://creativecommons.org/licenses/by/4.0/).

experiments than when exposed to either vocalizations or wingbeats alone. They also test for differences depending on whether the sounds come from a predator or a non-predator.

This is an interesting and compelling study, but several points would benefit from clarification, particularly regarding wingbeat stimuli used in the playbacks, and the model structure, including checks for overfitting and the specification of the random effects. Specifically:

Comment: * Where do the wingbeat sounds come from, and how far was the flying animal from the recording equipment?

Response: We obtained the wingbeat sounds through personal communication from David J. Shearer & Elizabeth A. Beilke from the study “Playing it by ear: gregarious sparrows recognize and respond to isolated wingbeat sounds and predator-based cues” (https://doi.org/10.1007/s10071-022-01663-z). The predator wingbeat (sharp-shinned hawk) was recorded as the hawk flew from one perch to another in the direction of the microphone (Sennheiser Model ME66) covering 10 m distance. The non-predator wingbeat (dark-eyed junco) was recorded as the junco departed from a feeding platform with generally 0.5 m from the microphone (Sennheiser Model ME66). We have included the information in the relevant Methods section. Acoustic Stimuli & Experimental design. See lines 168-173.

Comment: * Even though the experiment was conducted in captivity, the setup should reflect naturalistic conditions. Decibel level itself could affect the response of the animals (e.g. louder than in nature?)

Response: Yes. The loudness of the stimuli could affect the anti-predator response with lower response to soft stimuli and higher response to loud stimuli. Because both vocalization and wingbeat were used as stimuli, we chose relatively loud wingbeat stimuli which could be unnatural. From this study, we can not conclude if softer wingbeat could have different expected response. We would require more control and experimental groups to answer the question. We have included the limitation in the discussion section. See lines 436-439, and lines 149-153.

Abstract:

Comment: -It could be slightly restructured. Lines 30-31 seem somewhat disconnected and could be blended. The experimental design (predator vs non-predator playback) should be introduced before presenting the results.

Comment: -Line 41: It is unclear what "temporal" refers to.

Response: Thank you for the suggestions. We have rephrased the Abstract.

Introduction:

Comment: - It would be helpful to specify in which species the cited evidence has been observed. It is not clear whether this refers only to birds or to other taxa as well.

Response: Yes, the cited evidence refers to birds. We have rephrased the section for clarity. See line 87-90.

Comment: - Is there any evidence that diurnal predators have evolved silent flight similar to some nocturnal birds? Do these predators typically approach prey in a glide, or is wingbeat noise common within a range where prey could hear it?

Response: Diurnal predators can glide during flight. Typically, hawks ambush prey and rely on surprise attacks through dense vegetation, and wingbeats can be heard by prey birds in small ranges. Though experimental evidence is limited, the inference is based on the biological and theoretical basis of threat perception. See Clark et al. 2020 (Evolution and Ecology of Silent Flight in Owls and Other Flying Vertebrates: https://doi.org/10.1093/iob/obaa001)

Methods:

© 2026. Published by The Company of Biologists under the terms of the Creative Commons Attribution License (https://creativecommons.org/licenses/by/4.0/).

Comment: - A brief note on the behavioral ecology of this species would be useful. Do they live in groups? How do they behave naturally in terms of predator avoidance? This could be mentioned in the introduction too.

Response: We have added the relevant information in the Introduction. See lines 104-109.

Comment: - Line 163: What is meant by "Wingbeat stimuli were obtained through personal communication"?

Comment: - Line 167: Where were the wingbeat recordings obtained?

Response: We have clarified this above and added the information in the manuscript. See lines 168-173.

Were the two wingbeat recordings matched in intensity? It is noted that wingbeat playbacks were presented at ~70-75 dB at perch level. Could the authors clarify whether this amplitude is biologically realistic for a predator's wingbeat as perceived by chickadees in nature? Playback at this intensity may be higher than naturally occurring wingbeat sounds and could elicit responses driven primarily by volume rather than the acoustic structure of the stimulus. A brief justification of the chosen amplitude or reference to natural wingbeat levels would strengthen confidence in the ecological validity of the results.

Response: The wingbeat recordings were matched for intensity. The playback wingbeat sounds could be louder than the naturally occurring wingbeat sounds. The louder stimuli were chosen because of expected response to loud stimuli (if any) as we did not manipulate loudness of the stimuli in this study like Shearer & Beilke (2023). This could be indeed a limitation for more naturalistic settings. We have added the explanations in methods (see lines 149-153) and in discussions (see lines: 436-439).

Comment: - If I understood correctly, the model includes a three-way interaction (condition × phase × playback type), resulting in 24 combinations with only 8 individuals per group. Could the authors clarify how they assessed potential overfitting and ensured the robustness of these interaction estimates?

Response: Yes, the inclusion of the three-way interaction (condition × phase × playback type) in the model is theoretically motivated. We expect the responses to change with an interaction of predator vs non-predator condition, baseline vs playback vs post-playback, and wingbeat-vocalization combination. As the reviewer correctly suggested, we can not reliably interpret the interaction with our small sample size. Thus, we have refrained from interpreting the interaction in the results but included the interaction term in the model for a conservative approach.

For robustness of the estimates, we have included weakly informative priors on all fixed effects (normal(0, 1)) on log scale for interaction coefficients and normal (0, 6) for the intercept which assume most effects are small or absent in the absence of data support, while still allowing the data to shift estimates toward large effects when evidence supports it.

Comment: - Line 220: Specifying "bird ID nested within phase" may misrepresent the repeated-measures structure, treating the same individual in different phases as separate units. This can underestimate within-individual dependence and inflate the effective sample size. Modelling bird ID as a random intercept (and optionally random slopes) would better account for repeated observations.

Response: Thank you for pointing this out. The model includes (1 + phase | ID) as random effect structure, which fits a random intercept and random slope for phase, both grouped by individual bird ID. We have rephrased this and added the model structure in the manuscript. See lines 236-238 and Supplementary Tables.

Discussion

© 2026. Published by The Company of Biologists under the terms of the Creative Commons Attribution License (https://creativecommons.org/licenses/by/4.0/).

Comment: -Line 354: It would be helpful to clarify whether predator and non-predator stimuli contain the same proportion of sound and silence. This includes not only the silent intervals between sounds but also how the sounds are structured overall. Providing spectrograms in the supplementary materials would be useful for both vocalizations and wingbeat sounds.

Response: We have included the spectrogram for all stimuli in the supplementary materials as figures.

Comment: -Additionally, could the authors comment on whether the non-predator wingbeat might be too similar to the wingbeats of chickadees themselves? This could influence how the birds perceive and respond to these stimuli.

Response: That's an interesting point. Yes, the dark-eyed juncos' wingbeat sounds can be more similar to the wingbeats of chickadees due to the similar body size. The behavioural response to acoustically similar wingbeat would be a great direction of research. We would guess the birds might respond in uncertainty to playback of wingbeat in isolation and more certain responses to context specific stimuli (e.g. chickadee call +wingbeat) as compared to solo wingbeat.

Figures:

Comment: Figures 2 and 3 have panels of different sizes, one with a title and one without. It would improve clarity and consistency to make the panels homogeneous, so they do not appear as two independent figures placed side by side.

Response: We have removed the title from the figures to be more consistent.

Comment: Reviewer 2: This manuscript explores the role of acoustic cues in predator detection in black-capped chickadees (Poecile atricapillus), with a focus on the potential integration of wingbeat and vocalization signals. Using playback experiments, the authors examine whether chickadees discriminate between predator and non-predator cues and whether temporal integration of acoustic information influences their responses. The study addresses an interesting and relevant question in the context of animal communication. Overall, the work has the potential to contribute to our understanding of within-modality cue integration, although several aspects of the experimental design and interpretation would benefit from further clarification.

Response: Thank you for your comments.

Comment: Main issues: I think the authors tend to interpret the lack of evidence to reject the alternative hypothesis (which could be due to several aspects, e.g. lack of power) as evidence of no difference in bird responses or that birds respond similarly. Also, I believe there are some interpretations that are not entirely supported by the statistics they have chosen. I think they should acknowledge this and draw conclusions with greater caution.

The paper presents the analyzed results using Bayesian statistics; therefore, the figures present predicted data. I think it will increase transparency if you include exploratory graph for raw data showing their natural dispersion as supplementary file. It would be great to have an equivalent figure to those of the main manuscript but showing true data spread.

Response: We have rephrased the conclusions where required as suggested throughout the manuscript. We have now included the exploratory graphs showing raw data in the supplementary. See Fig. S1, Fig. S2, and Fig. S3 in the supplementary.

Comment: L. 35-36 "…we found that chickadees did not respond more strongly to predator versus non-predator acoustic cues when presented individually". This result is an interpretation from data presented in Fig. 2 and 3. Credible intervals are wide, suggesting low precision. So, the lack of differences could be due to other reasons apart from birds not responding differently. I suggest being more cautious with the interpretation of the lack of differences and say something like: "…we found no differences in how chickadees responded to predator versus non-predator acoustic cues when presented individually".

© 2026. Published by The Company of Biologists under the terms of the Creative Commons Attribution License (https://creativecommons.org/licenses/by/4.0/).

Response: Thank you for your suggestion. We have rephrased the interpretation. See line 31-35.

Comment: L. 155 Could you add spectrograms of the stimuli you used? It would be useful for readers to know what range of frequencies these stimuli cover and what frequencies are predominant. It could be part of the supplementary data.

Response: We have added the spectrograms of all stimuli used in the supplementary. See Fig. S4 to Fig. S11.

Comment: L. 174 Explain what WO, VO, WV, and VW represent. In the previous paragraph it is mentioned what VP, VN, WP, and WN are, but they are not used elsewhere after that. Then, the authors introduce these new abbreviations without describing them after the first use. It is a bit confusing for the reader.

Response: Thank you for your suggestion. We have removed VP, VN, WP, WN and kept WO, VO, WV, VW abbreviations throughout.

Comment: L. 175 "each bird received all four playbacks" It is not clear what four playbacks you mean. It would be better for understanding the design to read the text in lines 181-186 first. For example, it could be placed right after mentioning WO, VO, WV and VW for the first time.

Response: We have rephrased accordingly. See lines: 184-186.

Comment: L. 177-180 Single-cue playbacks were always delivered before sequential playbacks. Therefore, the differences you find when you compare these groups might be due to arousal or sensitization. This should be more clearly stated in the discussion of the results. Although the authors mention this in the Methods section: "Single-stimulus presentations (WO and VO) were always administered prior to sequential presentations (WV and VW) … which could limit interpreting the results." this is not discussed elsewhere.

Response: We have added this point in the discussion. See lines: 362-364.

Comment: L. 189 It is not clear to me whether, during the 5-minute playback, the total duration of sound presented was the same regardless of whether it was a single-cue or sequential playback. In other words, if each audio lasted 1 minute, was the same audio played five consecutive times in the single-cue condition, while in the sequential condition one stimulus was played three times and the other twice (it is also unclear in which order)? Alternatively, was the single-cue condition structured as 1 minute of audio followed by 1 minute of silence, and so on, resulting in a total amount of sound that is half of that in the other condition? If the former is true, the differences among these stimuli (single vs. sequential) could be due to additive stimulation or increased acoustic exposure.

Response: Yes, during the 5-minute playback, the total duration of sound was similar for single-cue and sequential playbacks. The wingbeat-only stimuli were ~15 sec sound and ~45 sec silence, vocalization only stimuli were ~15-17 sec sound and ~45-43 sec silence, wingbeat-vocalization stimuli were ~16 sec sound and ~44 sec silence and vocalization-wingbeat stimuli were ~16 sec sound and ~44 sec silence. The spectrograms in the supplementary show the differences. The same 1 min audio file was played five consecutive times. We have added more clarification in the procedure section. See line 180-181 and supplementary Fig.S4 to Fig. S11.

Comment: L. 199 Did you test more than one bird per day? If so, could the starting time any effect on the responses (e.g. it might be that birds are more responsive early in the morning than in the afternoon)?

Response: Yes, we tested more than one bird per day. All birds were tested in the morning with a similar starting time 9:15 to 9:30. We have rephrased the sentence. See line 212

Comment: L. 210 Have you performed an inter-rater reliability test among your observers?

© 2026. Published by The Company of Biologists under the terms of the Creative Commons Attribution License (https://creativecommons.org/licenses/by/4.0/).

Response: While we did not calculate a formal inter-rater reliability scores across all observers and variables, we took several practical steps to ensure scoring accuracy. Vocalizations (number of *tseets*, number of *chick-a-de*e calls) were quantified with help of spectrograms of the audio recordings and the perch-hopping was extracted from the video files. Observers scrolled through sound files in Audacity and noted the number and type of vocalizations, essentially ensuring complete accuracy, as *tseets* and *chick-a-dee* calls are unambiguously recognizable and classified even by rank amateurs and not missed or misclassified by our trained observers. The same is applicable for perch hops tallied from videos. A hop is an unambiguous behaviour. PKS cross-checked the call counts and perch hop counts for 3-4 selected audio and video recordings (3-4 hours) (from different birds) measured by KN and JR and found them to be accurate. We excluded time spent near the speaker variable as agreement was poor between PKS and KN and JR. We have added this in the manuscript see lines 220-225.

Comment: L. 220-222 Did the hurdle model for the chick-a-dee calls only include playback type as an effect? Could you explain further why you did not include phase and bird ID, as in the other models?

Response: We tried running a similar model for chick-a-dee calls but could not optimize the model parameters to converge the hurdle model accurately. So, we decided to run a simplified model to obtain some results about the chick-a-dee calls instead of discarding the data. We have added this information in the methods see lines 238-242.

Comment: L. 223 Could you specify which priors were used.

Response: Priors on all fixed effects (normal(0, 1)) on log scale for interaction coefficients and normal (0, 6) for the intercept, student_t(3,0,3) for variance fixed effect. We have included this in line 244-246 and the code shows the model and analysis.

Comment: L. 227-233 I think it would be better to understand the results if you could elaborate on how to interpret Bayesian results. I know that Bayesian statistics are widely used in science, but in some disciplines, there are people not entirely with them yet. I believe it will improve the manuscript if you can provide a brief explanation of how to interpret evidence ratios, evidence for directional hypothesis, posterior probabilities, etc.

Response: Thank you for your suggestion. We have included a brief explanation in the Statistical Analyses section. See lines: 256-266.

Comment: L. 231 Why did you use both means and medians along the manuscript? Could you explain why you chose one over the other?

Response: We had reported mean estimates for the negative binomial models and medians for the hurdle model. We have modified the results and now included the means for all uniformity. See lines 253, 338-342 and figure 4.

Comment: L. 234 Could you provide the tables associated with the models you interpret? I wonder if it is appropriate to provide general data about predator vs non-predator groups when it is unclear whether interactions were significant or not.

Response: Given our small sample size we decided not to interpret the interactions but included them in the model as conservative approach. We have added the tables associated with the models in the supplementary.

Comment: L. 240-244 These results are a bit confusing. It states that birds did not respond differently across phases, yet it also says that playback and post-playback phases were 11% lower than baseline. If I understood correctly, the results show no differences among phases. It is ok to report the mean values, SD and range for each phase, but providing a magnitude of the difference when no difference is supported might lead to confusion. A similar situation occurs in L. 249-252, 285-288, and 293-294.

© 2026. Published by The Company of Biologists under the terms of the Creative Commons Attribution License (https://creativecommons.org/licenses/by/4.0/).

Response: Thank you for the suggestion. We have removed the reported magnitude of differences in those places and kept the mean, SD and range values for clarity. See lines: 273-275, 306-309.

Comment: L. 245-252 Are you combining predator and non-predator groups responses? I believe you should not, because you do not expect the same magnitude of anti-predator responses for both groups; however, please make it clear in the text which group you are analyzing

Response: Yes, for the playback types, both predator and non-predator groups are combined in the analysis. We wanted to look at generally how responses differ in sequential playbacks vs solo playbacks. We agree, we would expect a different magnitude of response for both groups. We had analysed the data for the playback types in predator group as compared to non-predator group, which showed a greater difference than what is reported in the manuscript. But here we focused on sequential vs solo playbacks as we wanted to simplify the results and did not observe differences in predator vs non-predator groups. We have added a clarification in line 276 and 310.

Comment: L. 249-251 "Birds responded with 30% more perch hops to combined playback types (VW and WV) compared to solo playback types (VO and WO) (evidence ratio = 2.37, posterior probability of increase = 70%)" Would not be more accurate to say that you found no differences between combined vs solo playback types, given that evidence ratio and probability?

Response: Thanks for the feedback. Yes, that would be accurate to say. We have rephrased this sentence and other similar sentences. Lines 281, 315.

Comment: L. 267-274 The interpretation of the results related to variability or dispersion in the responses seem somewhat ambiguous. It states that "Sequential combined playback types showed substantially higher dispersion parameter values than solo playback types, indicating less overdispersion response patterns during sequential presentations". I believe it might help to report the magnitude of these effects and provide back-transformed estimates that would improve interpretability.

Response: We have back transformed the values from the log scale to natural scale. We have also included the evidence ratio for the dispersion responses for interpretability. See lines 289 and 323.

Comment: L. 325-327 I believe this result should be presented with more caution. With a posterior probability of 88%, it would be more appropriate to refer to a tendency. I understand that threefold difference is large, but (1) you are working with small numbers (as noted in L. 323 "Approximately 70% of observations recorded zero chick-a-dee calls across all conditions"); and (2) only one of the sequential playbacks supports that difference, the other sequential playback (VW) is similar to the solo vocalization playback (median 0.8 vs 0.62).

Response: Thank you for flagging it. We have rephrased the results. See lines 333-335

Comment: L. 330 Please include the meaning of HPD in the main text.

Response: We have added the meaning in the text. See lines 338-344.

Comment: L. 350-352 Could you elaborate further on this preliminary analysis? I may have missed the part where you statistically compare wingbeat-vocalization sequence vs. the other conditions. Perhaps this could be included as a separate analysis in the supplementary material.

Response: This was based on the results from the preliminary hurdle analysis results described in Chick-a-dee calls results (Lines 330-344) and Figure 4. As the majority of responses were 0s for chick-a-dee calls and the full model did not converge, we called it preliminary analysis. We have added this result in the manuscript now see lines 342-344 and 355-358.

Comment: L. 369-370 "Our results suggest that wingbeat played before vocalization elicited higher response and had lower overdispersion." I do not think it is appropriate to draw this conclusion if the WV playback was not analyzed independently.

Response: We have rephrased this accordingly. See lines 363-368.

© 2026. Published by The Company of Biologists under the terms of the Creative Commons Attribution License (https://creativecommons.org/licenses/by/4.0/).

Comment: L. 371 "range included negative values" Could you back-transform the coefficients to aid interpretation?

Response: We have added the back-transformed coefficients and modified interpretation. See lines 289, 292-295, 323, 325-329, 381

Second decision letter

MS ID#: bio.062598R1

MS Title: Differential response variability of black-capped chickadees to wingbeat sounds and vocalizations

Authors: Prateek K. Sahu; Kailyn Nottebrock; Julian Ratch; Sarah Smeltz; Katharine Stenstrom; Moriah Deimeke; Christopher Sturdy

I am happy to tell you that your manuscript has been accepted for publication in Biology Open, pending our standard publication integrity checks. It was accepted on 4th June 2026.

© 2026. Published by The Company of Biologists under the terms of the Creative Commons Attribution License (https://creativecommons.org/licenses/by/4.0/).
